# Investigation of multi-drug resistant *Candida auris* using species-specific molecular markers in immunocompromised patients from a tertiary care hospital in Quetta, Pakistan

**DOI:** 10.1371/journal.pone.0319485

**Published:** 2025-04-24

**Authors:** Hira Ejaz, Muhammad Mushtaq, Shereen Khan, Nasir Azim, Abrar Hussain, Kaleemullah Kakar, Muhammad Zubair Khan, Ayisha Hafeez, Syed Moeezullah

**Affiliations:** 1 Department of Biotechnology, Faculty of Life Sciences & Informatics (FLS&I), Balochistan University of Information Technology, Engineering and Management Sciences (BUITEMS), Balili, Quetta, Balochistan, Pakistan; 2 Fatima Jinnah Chest Hospital, Quetta, Pakistan; 3 Department of Mathematics, Faculty of Basic Sciences, BUITEMS, Takatu Campus, Quetta, Balochistan, Pakistan; University of the Witwatersrand, SOUTH AFRICA

## Abstract

**Introduction:**

*Candida auris* is an emerging multidrug-resistant pathogen responsible for nosocomial infections worldwide, characterized by high mortality rates and significant challenges in detection due to frequent misidentification. Classified by the WHO as a pathogen of critical importance since it exhibits resistance to multiple antifungal agents, particularly fluconazole, and is highly transmissible in healthcare settings. Conventional detection methods often lack the accuracy required for effective infection control. This study aimed to conduct inferential and molecular analyses of *C. auris* and other yeast species infecting immunocompromised patients in the Special and Intensive Care Units (SCU and ICU) of a tertiary care hospital in Quetta, Pakistan. In this region, *C. auris* remains rarely studied and is frequently misdiagnosed by clinical staff due to limited awareness and diagnostic challenges. Notably, no prior research has been conducted on *C. auris* in Quetta. The study also sought to develop reliable diagnostic methods suitable for resource-limited settings, addressing a critical gap in healthcare infrastructure.

**Materials and methods:**

Samples (150 each) from the ear, axilla, groin, and saliva of SCU/ICU patients were collected and processed on yeast malt agar, with preliminary identification using Brilliance *Candida* Agar (BCA) and CHROMagar *Candida* Plus (CCP). Advanced techniques, including PCR amplification of ITS regions, DNA sequencing, RFLP with *Msp*1, MALDI-TOF, Vitek 2, and species-specific primers, were used for identification. Antifungal susceptibility to fluconazole, amphotericin B, and voriconazole were also assessed.

**Results:**

The culture test revealed that 42.6% samples were positive for yeast infections. In addition to detecting *Candida auris* in 4 cultures, chromogenic media identified 6 other *Candida* species: *C. albicans*, *C. dubliniensis*, *C. glabrata*, *C. krusei*, *C. parapsilosis*, and *C. tropicalis*. Further validation through advanced techniques, including molecular diagnostics and MALDI-TOF, enabled the identification of additional species: *C. famata*, *C. kefyr*, *C. lusitaniae*, and *Meyerozyma* (*Candida*) *guilliermondii*. Out of all identified yeast species *C. dubliniensis* was the most common, followed by *C. albicans* and *C. tropicalis*, with the highest infection rates observed in saliva samples. Antifungal Susceptibility Tests (AST) revealed that *C. auris* isolates were resistant to Fluconazole, Amphotericin B, and Voriconazole, highlighting multidrug resistance. This study represents the first report of novel multidrug-resistant *C. auris* from Quetta, Pakistan, indicating that *C. auris* is prevalent among ICU and SCU patients. Novel species specific primers targeting phospholipase B, topoisomerase II, CDR and 18s genes were designed in our laboratory and not previously reported in earlier studies, proved highly effective for the rapid identification of *Candida* species. The established protocol using these primers is recommended for implementation in resource-limited laboratory settings. The statistical analysis demonstrated significant correlations between *Candida* species infection (dependent variable) and several independent factors (variables) emphasizing the importance of targeted diagnostics and intervention strategies.

## Introduction

Out of almost 300 species in the genus *Candida*, several are known to be associated with humans, causing infections primarily in immuno-compromised persons [1]. These infections contribute significantly to morbidity and mortality, particularly in immunocompromised and long-term hospitalized patients. *Candida* exhibits virulence that enables it to infect individuals across all age groups, with infection ranging from moderate to severe. The overuse of broad-spectrum antibiotics has further exacerbated resistance among non-albicans species. Despite advancements in medical diagnostics and treatment, fungal infections remain challenging to diagnose and cure [2].

*Candida albicans* typically colonizes various anatomical sites without causing harm. However, environmental changes can lead to mucosal to invasive systemic infections, resulting in damage mediated by the host and the pathogen [3]. While *C. albicans* is the most frequent cause of yeast infections, other species such as *C. glabrata*, *C. tropicalis*, *C. krusei*, and *C. dubliniensis* are increasingly prevalent. Recently *Candida auris* has emerged as a significant fungal pathogen, characterized by multidrug resistance and ability to cause nosocomial infections globally [4–5].

It is estimated that over 1/3 of patients with invasive *C. auris* infections, such as those affecting the bloodstream, heart, or brain, succumb to the disease. During the COVID-19 pandemic, the Pan American Health Organization (PAHO) World Health Organization (WHO) issued an epidemiological alert on February 6, 2021, highlighting outbreaks of *C. auris,* particularly among ICU patients, who are at the highest risk [6]. By late 2020, seven countries, including Italy, the United States and in Latin America, reported cases of *C. auris* in patients affected by COVID-19 [7].

*Candida auris* was first identified in 2009 in Japan through ribosomal DNA (rDNA) sequencing from a patient’s ear. Retrospective analyses revealed its presence as early as 1996 in bloodstream infections in South Korea [8]. In 2014, an outbreak at Aga Khan University Hospital in Karachi, Pakistan, initially misidentified as *Saccharomyces cerevisiae*, was later confirmed as *C. auris* due to its unique antifungal resistance pattern. Subsequent reports from Venezuela, South Africa, India and United States have drawn significant attention [9–10].

The Quetta city lies in the under developed region (Balochistan) of Pakistan where the situation is compounded due both to inadequate diagnostic facilities and management of *Candida* infection, especially emerging novel *C. auris*. Despite the critical need for regional data, no prior studies have systematically assessed the occurrence of *C. auris* in this region, particularly among hospitalized immunocompromised patients. This research study aims to provide a comprehensive and accurate framework for identifying *C. auris* and other *Candida* spp. addressing diagnostic challenges employing a combination of traditional and advanced diagnostics techniques. The focus is on delivering a reliable approach for the identification of these species in immunocompromised patients at a tertiary care hospital in Quetta, Pakistan.

The pathogen *C. auris* demonstrates resistance to multiple antifungal drugs, with over 90% resistance to fluconazole and 73% resistance to Voriconazole. Although some strains show resistance to amphotericin B, echinocandins remain the most effective treatment. The pathogen is also challenging to identify using traditional methods, often leading to underreporting. Phylogenetic analyses suggest that *C. auris* originated independently in four regions: South Asia, East Asia, Africa, and South America [10].

Yeast identification commonly relies on morphological, biochemical and molecular characteristics [11]. Morphological methods include observation of budding cells, hyphal development, germ tube formation and colony features [12–13]. Chromogenic media, such as Brilliance CHROMagar, enable species differentiation through characteristics colony coloration [14]. Molecular diagnostics, particularly DNA-based techniques, provide precise and rapid identification, notably for species like *C. auris* [14–16]. PCR amplification of ITS regions using universal primers (e.g., ITS1–4) are effective for almost all fungal genera including *Candida* spp. Techniques such as PCR-RFLP and species specific primers targeting genes such as topoisomerase II, phospholipase (PLB), and *Candida* Drug Resistant (CDR) genes further enhance identification accuracy have been effective for the detection of *Candida* species [14,17–29]. Clinical laboratories may misidentify *C. auris* as other species such as *C. kefyr*, *C. famata or C. haemulonii*, using manual and automated commercial technologies based on phenotypic and biochemical features including Vitek 2, Phoenix, API 20C, and MicroScan, resulting in inadequate management of yeast infections [25–27].

On the other hand, specific primers which can amplify DNA fragments from genomic and hybrid DNA ensuring rapid and targeted species-level identification of *Candida* species [25,28–34]. The PLB gene known for its diversity, is particularly recommended for primer design due to its role in pathogenicity [35–38]. DNA topoisomerases are enzymes that facilitate topological alterations in DNA by transiently cleaving and reconnecting double-stranded DNA. The DNA topoisomerase II cleave and ligate both strands of DNA to alleviate supercoiling. The genes of such enzymes are strong enough targets for phylogenetic research and the identification of fungal species [39–40]. Similarly, CDR (*Candida* Drug Resistance) genes like CDR1–5 that facilitate efflux via ATP-binding cassette (ABC) transporters, a key mechanism in antifungal resistance, against azoles, and other potential drugs such as polyenes and echinocandins as compared to other genes, makes it a very potential gene for the targeted identification of *C. auris* and other *Candida* species [40–46].

Recent therapeutic advancements emphasize echinocandins and broad-spectrum azoles for managing candidemia, invasive candidiasis, and mucosal infections. Fluconazole, initiated with an 800mg loading dose followed by 400mg daily for at least 14 days after a negative blood culture, remains the standard treatment for most *Candida* infections [47–48]. Other antifungals, such as boric acid, nystatin, and flucytosine are used for specific cases including intravaginal applications. Amphotericin B is reserved for severe cases, such as oral thrush in HIV patients [49–50].

## Materials and methods

### Ethical approval of research study

This study was conducted with approval from the Institutional Review Board (IRB) approval under the protocol titled “Prevalence, analysis and molecular diagnosis of newly emerging multidrug resistant human pathogenic *Candida auris* in the population of Quetta”. Written informed consent was obtained from patients or their guardians using standardized consent forms, permitting sample collection for research purposes. Socio-demographic data were collected through a structured questionnaire which included personal information (bed number, ward, duration of admission etc.), clinical symptoms, comorbidities, ongoing treatments, and medication.

### Study design

**Fig1:**
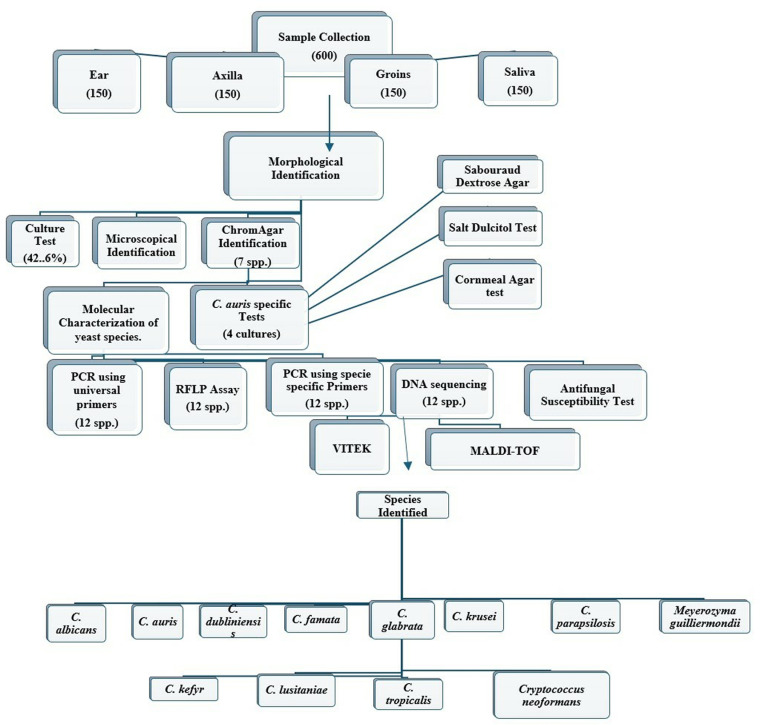
Flowchart of research study.

The study design involved the collection of clinical samples, followed by culture testing. An overview of the study design is illustrated in Fig 1. All cultures with growth were sequentially analyzed to differentiate yeast species using two chromogenic media. Identification was further confirmed through PCR amplification of ITS regions using universal primers (ITS1&4, ITS 1&2, and ITS3&4) followed by DNA sequencing. Additional techniques included PCR-restriction length polymorphism (PCR-RFLP) assays using the *Msp*1 restriction enzyme, MALDI-TOF analysis, Vitek 2 system, and species-specific primers targeting genes such as Phospholipase B (PLB), *Candida* Drug Resistance (CDR), Topoisomerase II, and 18S genes. The cultures of *Candida auris* were also tested for growth on salt Sabouroud agar (SDA), corn meal agar (CMA) and using salt Dulcitol Test. Identified yeast species underwent phylogenetic and statistical analysis. Antifungal susceptibility was assessed against fluconazole, amphotericin B and voriconazole.

### Sample collection

This study focused on immunocompromised patients admitted to the Special Care Units (SCU) and Intensive Care Units (ICU) of Fatima Jinnah Chest Hospital in Quetta, Pakistan. Patients were selected based on their clinical indicative presentation of symptoms (fever, chest infection, cough, ear infection etc.), indicative of yeast infections (comorbidities and treatment taken), hospital stay for (≥2 days), and their critical medical condition requiring specialized care. Samples were collected from multiple body sites (ear, axilla groin, oral cavities, etc.), as *Candida auris* is known to colonize various anatomical locations. From 21/10/2022 till 20/10/2023, swab samples were obtained from 150 immunocompromised patients admitted to ICU and SCU Fatima Jinnah Chest Hospital, Quetta, Pakistan. Each patient provided samples from the ear axilla, groin and saliva/sputum, regardless of age and gender.

For comparison, 100 swab samples from the ear, axilla and saliva were collected from healthy individuals, the comprehensive sampling strategy was implemented to capture colonization patterns and align with the study’s objectives of investigating *C. auris* prevalence, distribution and colonization across different body sites.

### Culture testing and yeast identification

Culture test was conducted on Yeast-Mold (YM) Agar medium, prepared using glucose (10 g), peptone (5 g), malt extract (3 g), yeast extract (3 g), and agar (20 g), these components were purchased separately and combined to prepare the medium. Swab samples were initially inoculated into pre-prepared YM Broth in glass culture tubes, incubated at 37 °C for 24–48 hours and subsequently transferred to YM agar plates using a sterile wire loop. Saliva/sputum samples were directly streaked onto YM agar plates and incubated at 37 °C for 24–48 hours, and subsequently transferred to YM agar plates under the same conditions.

Yeast growth was confirmed microscopically by preparing slides and observing the cells at 100× magnification using compound light microscope (Optika Microscopes, Italy) connected to a PC tablet [1].

### Chromogenic media identification

To differentiate yeast species, two chromogenic media were used such as, Brilliance CHROMagar (BCA) (OXOID) and CHROMagar *Candida* Plus (CCP) (CHROMagar^TM^) which is considered as an advanced medium with enhanced accuracy in distinguishing *Candida* spp. as described by [51]. All yeast species are identified based on colony color and appearance.

### Specific testing for *Candida auris*

Suspected *C. auris* isolates were grown on cornmeal agar as *C. auris* does not produce pseudohyphea [52]. To access its high salt and temperature resistance, isolates were tested on Salt Dulcitol test [53] and Salt Sabouroud test [54]. Due to the limitations of conventional methods, time consuming and less reliable, molecular techniques were employed for rapid and accurate identification. These techniques allowed for the precise validation of *Candida* species, particularly *C. auris.*

### Molecular characterization

The molecular characterization of isolated and purified yeast cultures was performed using PCR amplification. Three sets of universal ITS primers (ITS1&4, ITS1&2, ITS3&4) and species-specific primers targeting Phospholipase, Topoisomerase, CDR and 18S genes were employed. Species identification was validated through DNA sequencing of PCR products and PCR-Restriction Fragment Length Polymorphism (RFLP) assay using *Msp*1 Restriction enzyme [55]. Species-specific primers were designed using Primer3 software and optimized through *in-silico* PCR.

### DNA extraction and primer preparation

DNA was extracted using cetyl trimethyl ammonium bromide (CTAB) method and the quality was accessed via 1% gel electrophoresis. 20 µl of PCR reaction mix was prepared including 1.5 µl of each reverse and forward primers, 2µl template DNA. PCR reaction consisted of initial denaturation (95 °C for 6 mins), 40 cycles of cyclic temperatures included denaturation at 95°C for 40sec, annealing temperature (with respect to primers) for 40 sec and 72 °C for 1 min (extension) with a final extension at 72 °C for 5 min. The annealing temperatures for primers pairs ITS1&4, ITS1&2 and ITS 3&4 primers were, and respectively. Whereas the annealing temperatures for species-specific primers are summarized in Table 1.

**Table 1:** Primers designed for the identification of yeast species.

No.	Targeting spp.	Primers’ name		Sequence	Annealing temperature	Band size
**Universal ITS primers:**						
1.	Yeast spp.	ITS1&4	F	TCCGTAGGTGAACCTGCGG	59 °C	According to yeast species
			R	TCCTCCGCTTATTGATATGC		
		ITS1&2	F	TCCGTAGGTGAACCTGCGG	61.5 °C	
			R	GCTGCGTTCTTCATCGATGC		
		ITS3&4	F	GCATCGATGAAGAACGCAGC	58 °C	
			R	TCCTCCGCTTATTGATATGC		
**18S gene primers:**						
1	*Candida auris*	CAU (18S)	F	TATCACCGCAGATGTTTGGA	59.35 °C	167 bp
			R	CAAGAAGCTCGTAGGCAACC		
**PLB gene primers:**						
1.	*Candida albicans*	CA(PLB)	F	GGCTCATCTGGTGGAACATT	60.55 °C	164 bp
			R	TGGTACCATGAACTGCCTGA		
2.	*C. auris*	CAU(PLB)	F	CGTGAAAAACGACACCCTTT	59.4 °C	230 bp
			R	AGGTGTTTGGATCAGGAACG		
3.	*C. dubliniensis*	CD(PLB)	F	CTCAAGGCTTGTGGGAACTC	60.35 °C	225 bp
			R	TGGTTACATCGGACCACAAA		
4.	*C. famata*	DH(PLB)	F	TGAAGCAAAACAGGATGCAG	58.95 °C	193 bp
			R	CGTTCCAGGTGTTCTTCCAT		
5.	*C. glabrata*	CG(PLB)	F	GCATGTGCCATCATGAAAAG	58.4 °C	160 bp
			R	CCTCTGGGTTGATGAAGGAA		
6.	*C. kefyr*	CKE(PLB)	F	CCCAGAGAAGTCCTCGACTG	61.6 °C	168 bp
			R	GCAGTAGCAATCCCACCAAT		
7.	*C. krusei*	CKR(PLB)	F	ACGGGTATTTCTGGTGTTGC	60.15 °C	198 bp
			R	CGGTGAGAGTGGGACATTTT		
8.	*C. lusitaniae*	CL(PLB)	F	GCCGATAAAATCTCCGATGA	58.15 °C	177 bp
			R	TCCTCCGGAGAATGCAATAC		
9.	*C. parapsilosis*	CP(PLB)	F	AACACGTTGTGGCAAATTCA	57.85 °C	234 bp
			R	TTGGAAACCGTTTTGAGACC		
10.	*C. tropicalis*	CT(PLB)	F	ATGTTGAATGGTGCTGGTCA	59.2 °C	230 bp
			R	TTCCAACCACCTGGATTCAT		
11.	*M. guilliermondii*	MG(PLB)	F	TGCGATGCTTCGAATATGAC	56.7 °C	161 bp
			R	CCCCATTTTTCATCGTTGTC		
12.	*Cryp. neoformans*	CRN(PLB)	F	ACTGCTGGTTTGGGCTTGAT	61.2 °C	200 bp
			R	CAATGCTGTGGAGCATGCAG		
**Topoisomerase-II gene primers:**						
1	*C. auris*	CAU(TOP)	F	TGGTTGAGCAGTTGATCGAG	58.85°C	209 bp
			R	GATGTTTTCCGGTTTGTGCT		
2	*C*. *parapsilosis*	CP(TOP)	F	CTGCGTATCAAGGGTCAGGT	58.6°C	220 bp
			R	CAGCGTTCTTTGCAATTTGA		
3	*C*. *glabrata*	CG(TOP)	F	TCTTCCGGTCTTGCTTCAGT	60.55°C	160 bp
			R	TTCCTGCACAAGCAAGTGTC		
4	*C*. *lusitaniae*	CL(TOP)	F	CAAGGACCACCGTTTCTGTT	60.45°C	219 bp
			R	CAGACAGCGCCTTATTCTCC		
***Candida* drug resistance (CDR) gene primers:**						
1	*C. dubliniensis*	CD(CDR)	F	TCTCACGTTGCCAAACAATC	57.65°C	196 bp
			R	CAGCAAAGAACATGGAAGCA		
2	*C*. *glabrata*	CG(CDR)	F	ACGGTACCAAGCCATACGAG	62.3°C	188 bp
			R	GAACACTGGGGTGGTCAAGT		
3	*C*. *auris*	CAU(CDR)	R	TGGTTACATCGGACCACAAA	62.5°C	164 bp
			F	TGAAGCAAAACAGGATGCAG		
4	*C*. *parapsilosis*	CP(CDR)	F	CTTCCGGTCACTTGAATGGT	60.15°C	221 bp
			R	TCCTCCATAATGGGCTTGTC		
5	*C*. *tropicalis*	CT(CDR)	F	GATCGGGAATTGCTCACACT	57.8°C	163 bp
			R	AATTTGCAGCCGTCAAAAAC		

### Yeast identification using VITEK and MALDI-TOF

The automated VITEK 2 Compact (bioMérieux, France) and MALDI-TOF mass spectrometry (Bruker Daltonics, Germany) were used to carry out phenotypic and biochemical identification of the yeast isolates that were selected for the investigation. All isolates were first cultured on YM Agar and incubated for 24–48 hours at 37 °C to ensure pure and viable colonies. A single colony from each culture was suspended in sterile saline to reach a turbidity of 0.5 McFarland for Vitek analysis. The Vitek 2 yeast identification cards (YST ID cards), which included pre-defined biochemical substrates, were loaded with the produced solutions. The technology provided species-level identification via database matching by automatically recording and analyzing metabolic profiles.

Each isolate was identified using MALDI-TOF by spotting one colony onto a target plate. One microlitre of matrix solution (α-cyano-4-hydroxycinnamic acid in 50% acetonitrile and 2.5% trifluoroacetic acid) was applied to the samples, and they were then left to air dry. After that, the target plate was put into the MALDI Biotyper apparatus. Protein mass fingerprinting was used to create spectral profiles, which were then matched to reference databases to identify species.

### Antifungal susceptibility testing

Antifungal susceptibility testing was performed using the disk diffusion method also known as Kirby-Bauer test, a standardized approach for evaluating antifungal resistance. This method involves placing paper disks impregnated with specific antifungal agents on agar plates inoculated with yeast cultures. As the antifungal agent diffuses during incubation, it creates a concentration gradient, resulting in zone of inhibition where fungal growth is suppressed. These zones of inhibition were measured and compared to standardized guidelines (CLSI) to determine antifungal susceptibility, i.e., susceptible, intermediate, or resistant (Table 2). Eleven *Candida* species were tested against fluconazole (5–25 µg/ml), amphotericin B (0.5–4 µg/ml), and voriconazole (0.5–4 µg/ml). Samples were first incubated in YM broth for 24 hours in a shaking incubator, diluted tenfold, and then inoculated onto YM agar plates using spread plate method. For each *Candida* species, 12 plates were prepared three controls and three replicates for each antifungal agent. Five sterilized disks with specific drug concentrations were placed on each plate, which were incubated for 37 °C for 48 hours. The inhibition zones were measured in millimeters (mm) to access the antifungal susceptibility of the isolates.

**Table 2:** Assessment of antifungal susceptibility as per standard guideline.

No.	Antifungal Drug	Interpretation of zone diameters		
Resistant	Intermediate	Susceptible
1.	Fluconazole	≤14 mm	15–18 mm	≥19 mm
2.	Amphotericin B	≤12 mm	13–15 mm	≥16 mm
3.	Voriconazole	≤13 mm	14–16 mm	≥17 mm

### Statistical analysis

The clinical data obtained from patients and the identification of yeasts species were statistically analyzed to access the significance of the results and to explore relationships between various variables using IBM SPSS 22. The frequency distribution of identified yeast species was calculated through crosstab analysis, Binary and multinomial logistic regression analyses were performed to examine the relationships between the dependent variable (yeast infection) and independent variables, including age gender, ward type, sample type, health disorders and symptoms. In multinomial regression the yeast species identified using ITS technique were selected as dependent variable with *C. albicans* as the reference category.

## Results

### Description of samples

The demographic and clinical characteristics of the patients, including gender, age, ward type, sample type, health disorders and symptoms were documented and summarized in Table 3, presented as numbers and percentages.

**Table 3:** The sociodemographic and clinical characteristics of patients under study.

	Sociodemographic and clinical characteristics	N (150)	Percentage
**Gender**			
	Male	64	42.66%
	Female	86	57.33%
**Age groups**			
	01–09	1	0.66%
	10–19	5	3.33%
	20–29	9	6%
	30–39	3	2%
	40–49	16	10.6%
	50–59	30	20%
	60–69	36	24%
	70–79	34	22.66%
	80–89	14	9.33%
	90–99	2	1.33%
**Ward**			
	ICU	111	74%
	SCU	39	26%
**Sample type**			
	Axilla	150	100%
	Ear	150	100%
	Groin	150	100%
	Saliva	150	100%
**Health disorder**			
	Diabetes	74	49.5
	Hypertension	76	50.8
	Covid-19	39	26.2
	Chest infection	45	30.3
	Ear infection	32	21.5
	Heart diseases	13	8.7
	Pneumonia	8	5.3
	Asthma	12	8.2
	Tuberculosis treated	11	7.1
**Symptoms**			
	Fever	141	94%
	Chills	81	54.1
	Ear itching	19	12.5
	Ear pain	30	20.2
	Blood in sputum	24	16.1
	Weakness	116	77
	Shortness of breath	78	52.3

### Morphological grouping of fungal cultures

Out of 600 samples collected from axilla, ear, groin and saliva of 150 patients, 256 samples tested positive for yeast on YM agar medium. The highest occurrence was observed in saliva samples (107/150), followed by ear (72/150), axilla (42/150) and groin (35/150). Morphological characteristics of yeast cultures were accessed both macroscopically and microscopically. Most colonies exhibited a glossy surface with smooth margins, and their colors varied from white to cream and pink after incubation after 48 hours of incubation at 37°C on YM agar.

Some samples indicated mixed yeast infection. Microscopically, most yeast cultures displayed round, spherical, or oval cells, while others produced filamentous structures such as hyphae or pseudohyphea. In contrast, 19 yeast cultures were isolated identified from 100 healthy individuals. Among these, 16 cultures produced white or cream-colored colonies and 3 yielded pink yeast colonies.

### *Candida* species identification on chromogenic media

**Fig 2:**
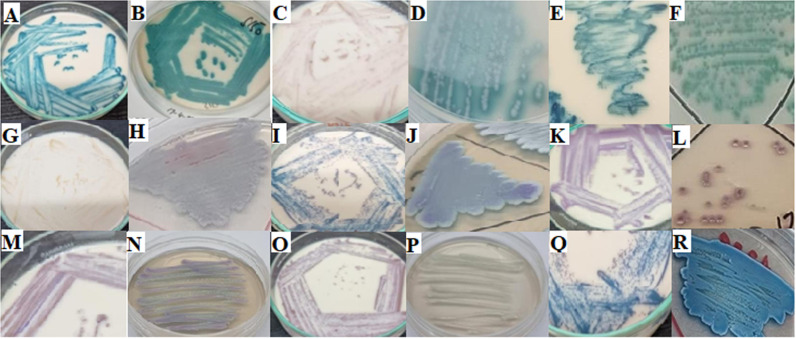
Colony color variations of Candida species on two different chromogenic media: (A) Candida albicans (BCA), (B) Candida albicans (CCP), (C) Candida auris (BCA), (D) Candida auris (CCP), (E) Candida dubliniensis (BCA), (F) Candida dubliniensis (CCP), (G) Candida glabrata (BCA), (H) Candida glabrata (CCP), (I) Candida kefyr (BCA), (J) Candida kefyr (CCP), (K) Candida krusei (BCA), (L) Candida krusei (CCP), (M) Candida lusitaniae (BCA), (N) Candida lusitaniae (CCP), (O) Candida parapsilosis (BCA), (P) Candida parapsilosis (CCP), (Q) Candida tropicalis (BCA), (R) Candida tropicalis (CCP).

All yeast cultures producing white or cream-colored colonies on YM agar medium were further tested using two different chromogenic media which revealed distinct colony colors and morphologies. Based on the colony appearances on BCA and CCP, these cultures were classified into seven different *Candida* species. The sensitivity and specificity of the chromogenic media were particularly high for identifying *C*. *tropicalis* and *C*. *albicans* as illustrated in Fig 2.

### Molecular characterization of yeast species

The molecular characterization of all isolated yeasts species was conducted using molecular identification and validation techniques. Yeast cultures were identified through PCR amplification of their Internally Transcribed Spacer (ITS) regions, employing three different primers sets: ITS1&4, ITS1&2, and ITS3&4. The results were validated through Restriction Fragment Length Polymorphism (RFLP) assay using *Msp*I restriction enzyme. Further validation included PCR amplification with species-specific primers designed for various *Candida* species, targeting such as Phospholipase B (PLB), Topoisomerase-II (TOP), *Candida* Drug Resistance (CDR), and 18S. Additionally DNA sequencing and phylogenetic analysis were performed to confirm species identification.

#### Identification through PCR amplification of ITS regions.

The standard PCR amplification technique was used to amplify the Internal Transcribed Spacer (ITS) regions of each yeast culture, employing three different sets of primers: ITS1&4, ITS1&2 and ITS3&4. The number of identified species in total number of participants is summarized in Table 4.

**Table 4:** Yeasts species identified through PCR amplification and sequencing of ITS regions isolated from patients and participants from healthy community.

No.	Yeast species	Patients	Healthy	Total
**1.**	*Candida albicans*	54	9	63
**2.**	*Candida auris*	4	0	4
**3.**	*Candida dubliniensis*	67	0	67
**4.**	*Candida famata*	5	0	5
**5.**	*Candida glabrata*	11	0	11
**6.**	*Candida kefyr*	19	0	19
**7.**	*Candida krusei*	24	0	24
**8.**	*Candida lusitaniae*	21	0	21
**9.**	*Candida parapsilosis*	10	0	10
**10.**	*Candida tropicalis*	40	4	44
**11.**	*Meyerozyma guilliermondii*	1	0	1
**12.**	*Cryptococcus neoformans*	0	3	3
	**Total**	**256**	**16**	**474**

**Fig 3:**
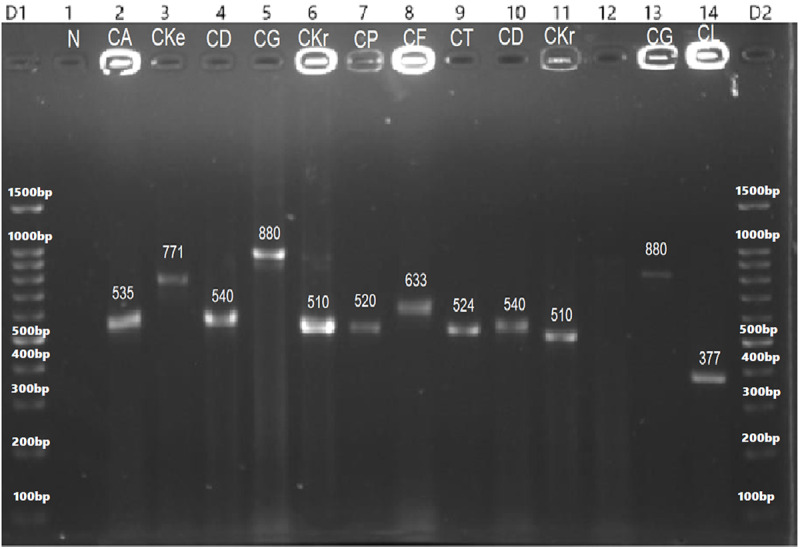
PCR amplified products using ITS1&4 primers of ITS regions of yeast species: 2= C. albicans (535 bp), 3= C. kefyr (771 bp), 4= C. dubliniensis (540 bp), 5= C. glabrata (880 bp), 6= C. krusei (510 bp), 7=C. parapsilosis (520 bp), 8= C. famata (633 bp), 9= C. tropicalis (524 bp), 10= C. dubliniensis (540 bp), 11= C. krusei (510 bp), 12 shows no result, 13= C. glabrata (871 bp), and 14= C. lusitaniae (377 bp) as compared to negative control (1) and 100 bp DNA ladder (D1 and D2).

**Fig 4:**
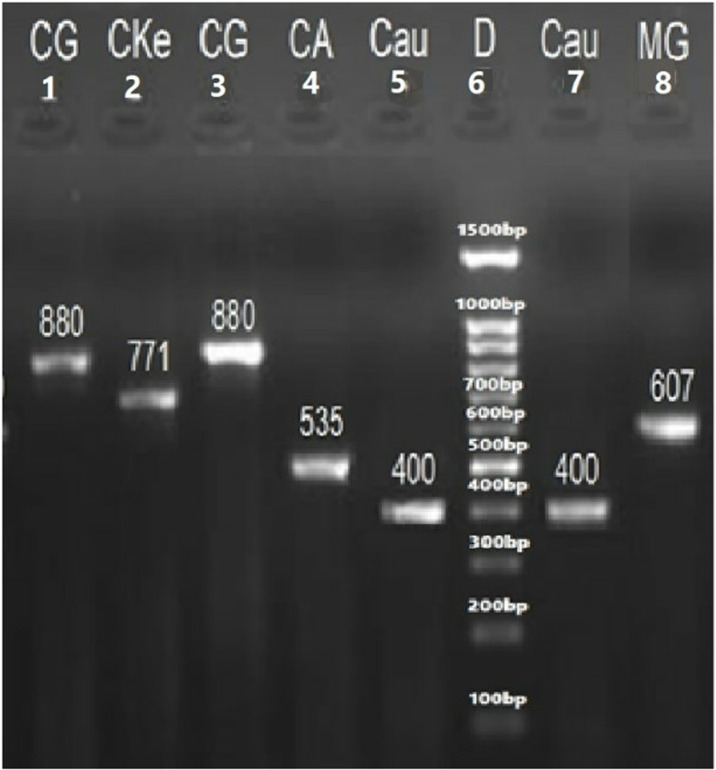
PCR amplified products of ITS1&4 primers of yeast species., 1= *C. glabrata* (871 bp), 2= *C. kefyr* (771 bp), 3= *C. glabrata* (871 bp), 4= *C. albicans* (535 bp), 5&7*= C. auris* (400 bp), 8*= Meyerozyma guilliermondii* (607 bp), and 6= 100 bp DNA ladder.

**Fig 5:**
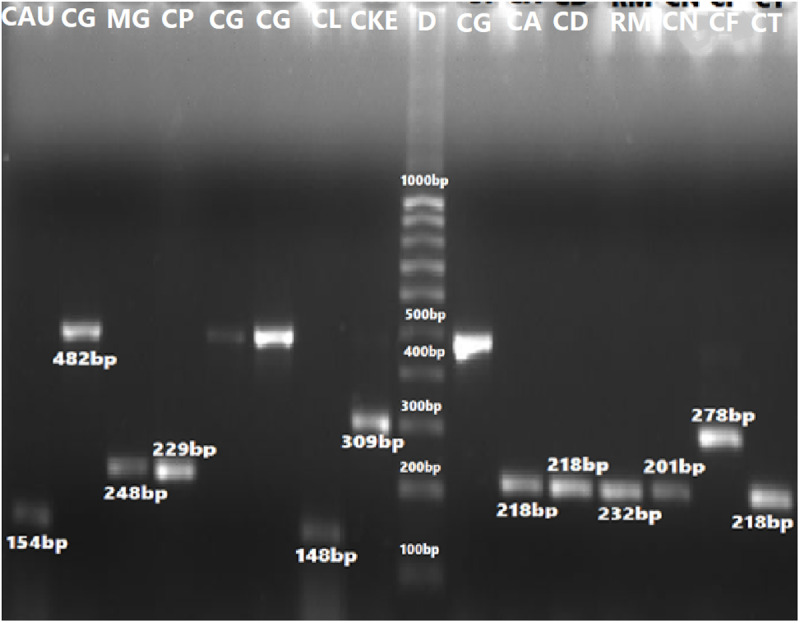
PCR amplified products using ITS1&2 primers of ITS regions of yeast spp., CAU= *Candida auris* (154 bp), CG= *C*. *glabrata* (482 bp), MG= *Meyerozyma guilliermondii* (248 bp), CP*= C*. *parapsilosis* (229 bp), 7= *C*. *lusitaniae* (148 bp), 8= *C*. *kefyr* (309 bp), 9= *C*. *albicans* (218 bp),10= *C*. *dubliniensis* (218 bp), 12= *Cryptococcus neoformans* (201 bp) 13= *C.famata* (278 bp) and 14*= C. tropicalis* (218 bp).

**Fig 6:**
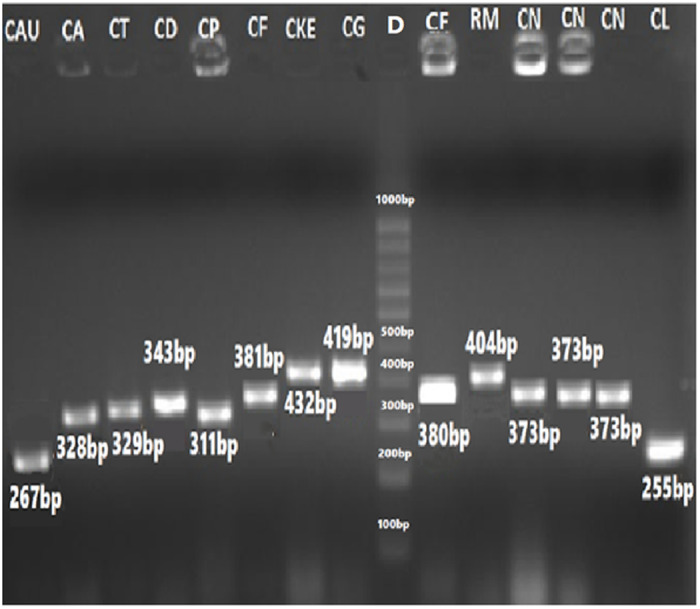
PCR amplified products using ITS3&4 primers of ITS regions of yeast species. CAU= *Candida auris* (267 bp), CA= *C*. *albicans* (328 bp), and CT=*C*. *tropicalis* (329 bp), CD= *C*. *dubliniensis* (343 bp), CP= *C*. *parapsilosis* (311 bp), CF= *C*. *famata* (381 bp), CKE= *C*. *kefyr* (432 bp) CG= *C*. *glabrata* (419 bp), CF= *C*. *famata* (381 bp), RM= *Rhodotorulla mucilaginosa* (404 bp) CN= *Cryptococcus neoformans* (373 bp) CL= *C*. *lusitaniae* (255 bp) as compared to 100 bp DNA ladder (9).

The sizes of their PCR amplified products obtained using the ITS1&4, ITS1&2 and ITS3&4 primers are summarized in Table 5. The visualized PCR products of ITS1&4 are illustrated in Figs 3 and 4, while those amplified with ITS1&2 and ITS3&4 primers are illustrated in Figs 5 and 6, respectively.

**Table 5:** PCR product sizes of different yeast species amplified using 03 different sets of primers, i.e., ITS1&4, ITS1&2 and ITS3&4.

No.	Name of yeast species	Band Size of PCR amplified products on 1.5% gel		
		ITS1&4	ITS1&2	ITS3&4
**1.**	*Candida albicans*	536	218	338
**2.**	*Candida auris*	401	154	267
**3.**	*Candida dubliniensis*	541	218	343
**4.**	*Candida famata*	639	278	381
**5.**	*Candida glabrata*	881	309	432
**6.**	*Candida kefyr*	720	182	347
**7.**	*Candida krusei*	509	482	419
**8.**	*Candida lusitaniae*	383	148	255
**9.**	*Candida parapsilosis*	520	229	311
**10.**	*Candida tropicalis*	525	218	327
**11.**	*Cryptococcus neoformans*	555	248	373
**12.**	*Meyerozyma* (*Candida*) *guilliermondii*	607	232	380

#### Validation through restriction fragment length polymorphism (RFLP).

**Fig 7:**
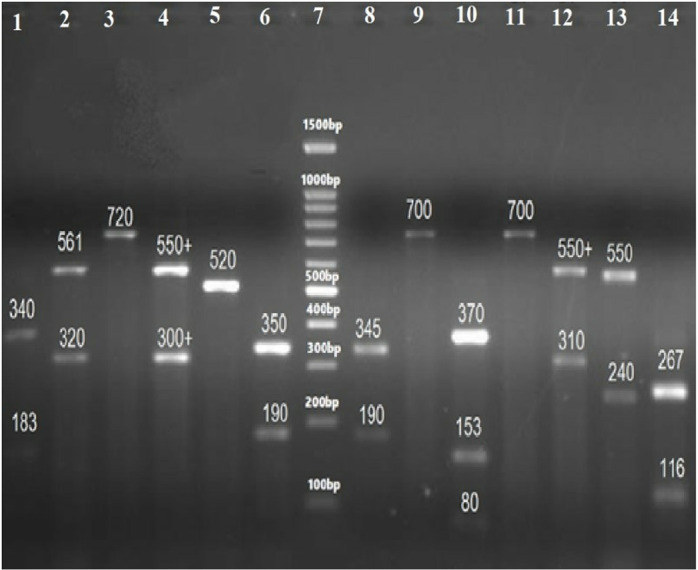
Gel image of PCR-RFLP Assay of ITS1&4 primers amplified products using Msp*1* as a Restriction enzyme. 1= *Candida tropicalis* (340,186 bp), 2, 12, 13= *C*. *glabrata* (320, 561 bp), 3, 9, 11= *C*. *kefyr* (771 bp), 4= *C*. *parapsilosis* (530 bp), 5= *C*. *dubliniensis* (340,200 bp), 6= *C*. *dubliniensis* (340, 200 bp), 7= *Meyerozyma guilliermondii* (82, 155, 370 bp) 8= *C*. *lusitaniae* (250,120 bp), and as compared to 100 bp DNA ladder (7).

**Fig 8:**
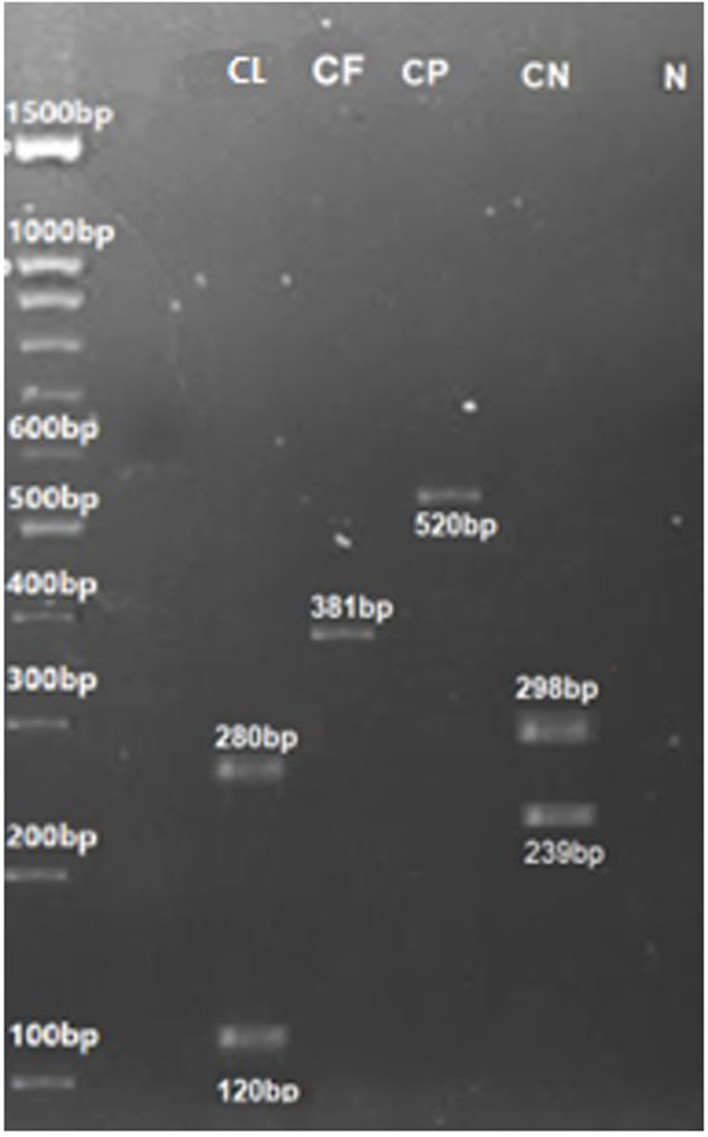
Digestion of PCR products of *Candida* species with ITS1-4 primers. Using Msp1 restriction enzyme, CL= Candida lusitaniae (267,116 bp), CF= C. famata (381 bp), CP= C. parapsilosis (520 bp), CN= Cryptococcus neoformans (239,298 bp) 100 bp DNA ladder and N = Negative Control.

**Fig 9:**
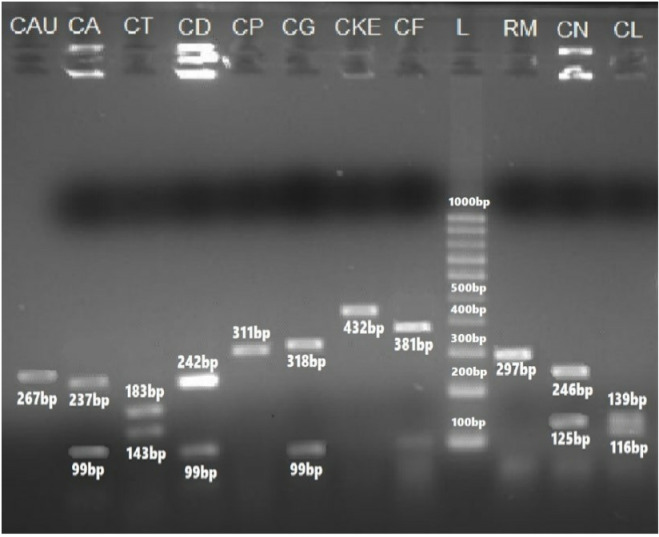
Gel image of PCR-RFLP assay using *Msp*1 as a restriction enzyme for the digestion of PCR products ITS3&4 of CAU= *Candida auris* (267 bp), CA= *C*. *albicans* (237 bp, 99 bp), and CT=*C*. *tropicalis* (183 bp, 143 bp), CD= *C*. *dubliniensis* (244 bp, 99 bp), CP= *C*. *parapsilosis* (350 bp, 200 bp), CG= *C*. *glabrata* (318 bp, 99 bp), CKe= *C*. *kefyr* (432 bp) CF= *C*. *famata* (381 bp), CN= *Cryptococcus neoformans* (246 bp, 125 bp) CL= *C*. *lusitaniae* (139 bp, 116 bp) as compared to 100 bp DNAladder (L).

PCR-based identification of *Candida* species was further validated using the Restriction enzyme *Msp*1 to Perform Restriction Fragment Length Polymorphism (RFLP) analysis. Band sizes were determined by downloading the reference sequences from NCBI and performing *in silico* digestion of nucleotide sequences using NEB cutter software. This allowed the calculation of DNA digestion products generated by the *Msp*1 enzyme. The sizes of PCR products and their corresponding digested fragments are summarized in Table 6 (Figs 7–9).

**Table 6:** PCR-RFLP assay of different PCR amplified products of ITS regions of yeast species using *Msp*1 restriction enzyme.

No.	Yeast species	PCR products of ITS regions using different ITS primers and their RFLP products					
		ITS1&4	RFLP pattern	ITS1&2	RFLP pattern	ITS3&4	RFLP pattern
**1.**	*Candida albicans*	536	297, 237	218	218	338	99, 237
**2.**	*C*. *auris*	401	401	154	154	267	267
**3.**	*C*. *dubliniensis*	541	297, 242	218	218	343	99, 242
**4.**	*C*. *famata*	639	639	278	278	381	381
**5.**	*C*. *kefyr*	720	720	309	309	432	432
**6.**	*C*. *krusei*	509	262, 247	182	182	347	100, 47
**7.**	*C*. *glabrata*	881	561, 318	482	482	419	99, 318
**8.**	*C*. *lusitaniae*	383	267, 116	148	148	255	139,116
**9.**	*C*. *parapsilosis*	520	520	229	229	311	311
**10.**	*C*. *tropicalis*	525	340, 183	218	218	327	142, 183
**11.**	*Cryptococcus neoformans*	555	428, 125	201	201	373	246, 125
**12.**	*Meyerozyma* (*Candida*) *guilliermondii*	607	370, 153, 80	248	248	380	142, 153, 80

### Validation through species-specific primers

#### Phospholipase-B (PLB) gene.

**Fig 10:**
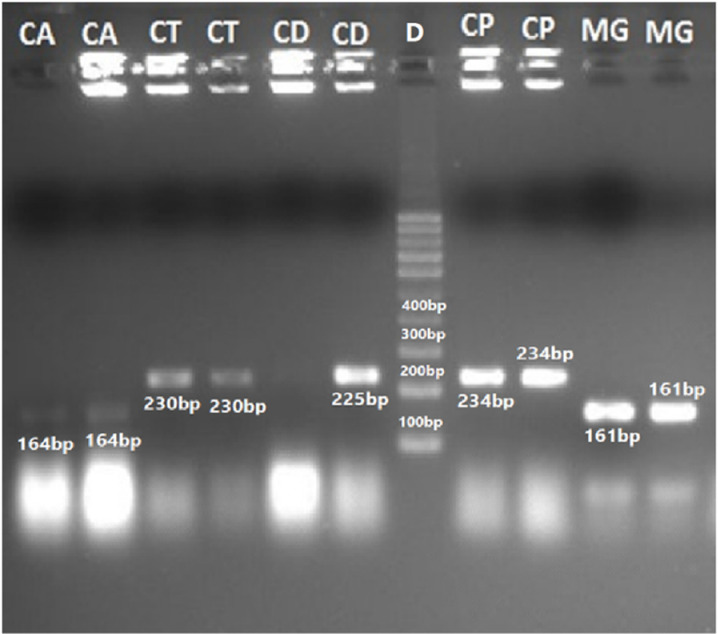
Using PLB gene species-specific primers PCR amplified product of CA=C. albicans (164 bp), CT=C. tropicalis (230 bp), C. dubliniensis (225 bp), C. parapsilosis (234 bp), Meyerozyma guilliermondii (161 bp) and 100 bp DNA ladder (D).

**Fig 11:**
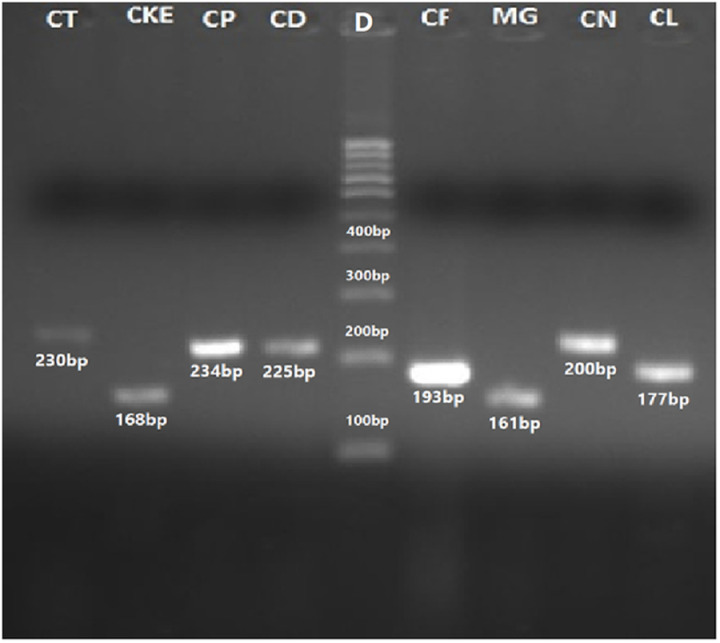
PCR amplified products of the PLB gene through specie-specific: CT = Candida tropicalis (230 bp), CKE = C. kefyr (168 bp), CP = C. parapsilosis (234 bp), CD = C. dubliniensis (225 bp), CF= C. famata (193 bp), MG= Meyerozyma guilliermondii (161 bp), CN= Cryptococcus neoformans (200 bp) CL= C. lusitaniae (177 bp) and D= 100 bp ladder (5).

**Fig 12:**
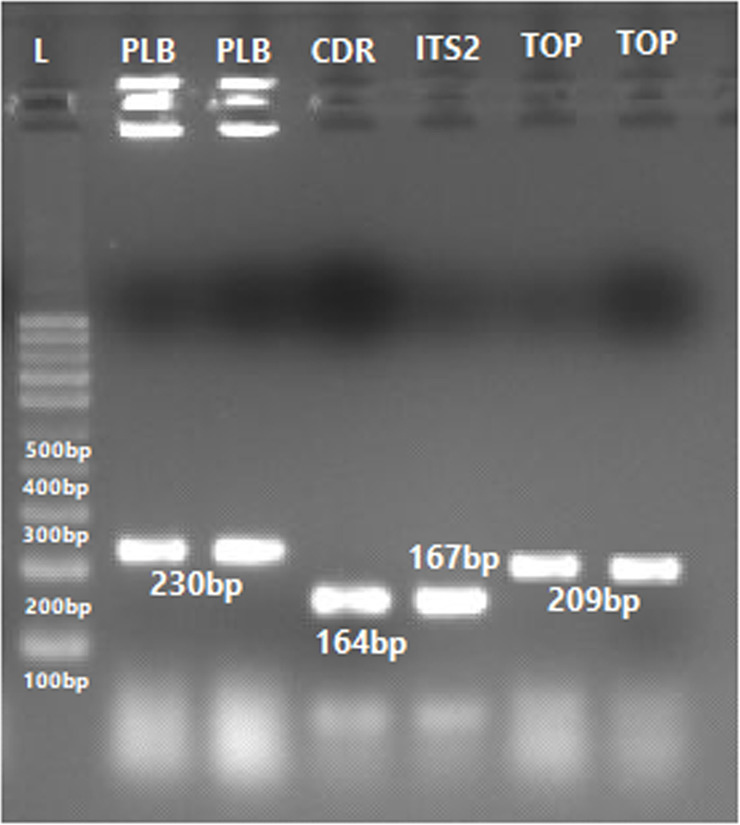
PCR amplified products of the Candida auris PLB= 230 bp, CDR=164 bp, 18S= 167 bp and TOP= 209 bp using PLB, CDR, 18S and Topoisomerase II (TOP) gene through specie specific primers.

The identification of all yeast species was further validated using species-specific primers. DNA sequences in FASTA format for the Phospholipase B (PLB) gene, Topoisomerase-II and *Candida* Drug Resistance genes were retrieved from NCBI, and primers were designed using Primer 3 online software. Species-specific primers for the Phospholipase B gene successfully identified *Candida albicans*, *C*. *dubliniensis*, *C*. *tropicalis*, *C*. *glabrata*, and *C*. *lusitaniae*. The amplified PCR bands were observed at 164 bp, 225 bp, 160 bp, 177 bp, 234 bp, and 230 bp corresponding to *C. albicans*, *C*. *dubliniensis*, *C*. *glabrata*, *C*. *lusitaniae*, *C*. *parapsilosis* and *C*. *tropicalis*, respectively (Figs 10–12).

#### Topoisomerase II (Top) gene.

**Fig 13:**
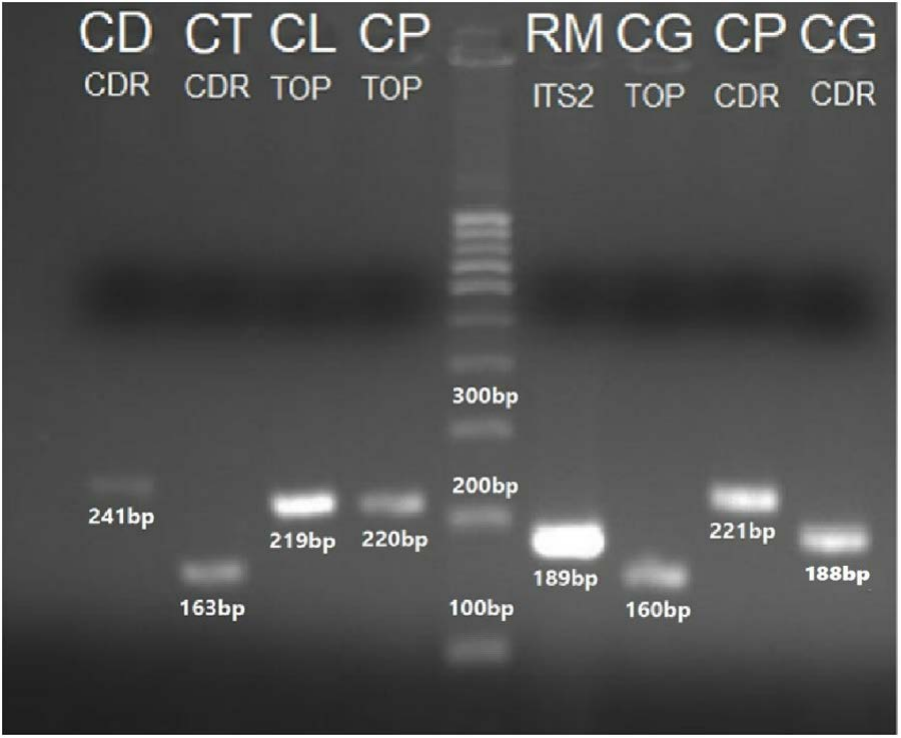
PCR amplified products through specie-specific primers of Topoisomerase II gene: CL(TOP) = *C*. *lusitaniae* (219 bp), CP(TOP) = *C*. *parapsilosis* (220 bp), CG(TOP) = *C*. *glabrata* (160 bp); CDR gene: CD(CDR)= *C*. *dubliniensis* (241 bp), CT(CDR)= *C*. *tropicalis* (163 bp), CP(CDR)= *C*. *parapsilosis* (221 bp), CG(CDR)= *C*. *glabrata* (188 bp); and D=100 bp ladder.

The identification of *Candida auris* (Fig 12), *C*. *glabrata*, *C*. *lusitaniae* and *C*. *tropicalis*, was further validated using species-specific primers targeting the Topoisomerase-II gene. The amplified PCR bands for these *Candida* species were observed at 209 bp, 219 bp, 220 bp and 160 bp, respectively (Fig 13).

#### *Candida* Drug Resistance (CDR) gene.

The identification of *Candida auris* (Fig 12), *C*. *dubliniensis*, C. *glabrata*, *C*. *parapsilosis* and *C*. *tropicalis* was also validated using species-specific primers targeting CDR gene. The amplified PCR bands were observed at 164 bp, 241 bp, 188 bp, 221 bp and 163 bp, respectively. These bands correspond to *C*. *glabrata*, *C*. *lusitaniae*, and *C*. *parapsilosis* (Fig 13).

#### 18S gene.

Species-specific primers targeting the 18S gene successfully amplified a PCR product of 167 bp, confirming the identification of *Candida auris* (Fig 12).

#### DNA sequencing and analysis of human pathogenic yeasts.

The ITS regions of all human pathogenic yeasts, visualized during PCR amplification and gel electrophoresis using universal primers, were sequenced. The obtained sequences of all yeast species were subjected to BLAST analysis against NCBI reference sequences, ITS, and non-redundant nucleotide databases. Based on BLAST search results, twelve (12) yeast species were identified: *Candida albicans*, *C*. *auris*, *C*. *dubliniensis*, *C*. *glabrata*, *C*. *guilliermondii*, *C*. *lusitaniae*, *C*. *kefyr*, *C*. *krusei*, *C*. *parapsilosis*, *C*. *tropicalis*, and *Cryptococcus neoformans*. All the Sequences were submitted to NCBI and accession numbers for each species listed in Table 7.

**Table 7:** Accession numbers of different yeast spp. received from NCBI after submission of DNA sequences.

No.	Yeast species	Submission code	Accession numbers
**1.**	*Candida albicans*	ICP_CA_ITS	PQ001768
**2.**	*C*. *auris*	ICP_CAU_ITS	PP994477
**3.**	*C*. *dubliniensis*	ICP_CD_ITS	PQ001769
**4.**	*C*. *famata*	ICP_CF_ITS	PP994643
**5.**	*C*. *kefyr*	ICP_CKE_ITS	PQ001770
**6.**	*C*. *krusei*	ICP_CKR_ITS	PP994677
**7.**	*C*. *glabrata*	ICP_CG_ITS	PP994490
**8.**	*C*. *lusitaniae*	ICP_CL_ITS	PP994497
**9.**	*C*. *parapsilosis*	ICP_CP_ITS	PP994522
**10.**	*C*. *tropicalis*	ICP_CT_ITS	PP994128
**11.**	*Cryptococcus neoformans*	ICP_CRN_ITS	PP994669
**12.**	*Meyerozyma* (*Candida*) *guilliermondii*	ICP_MG_ITS	PQ001771

**Fig 14:**
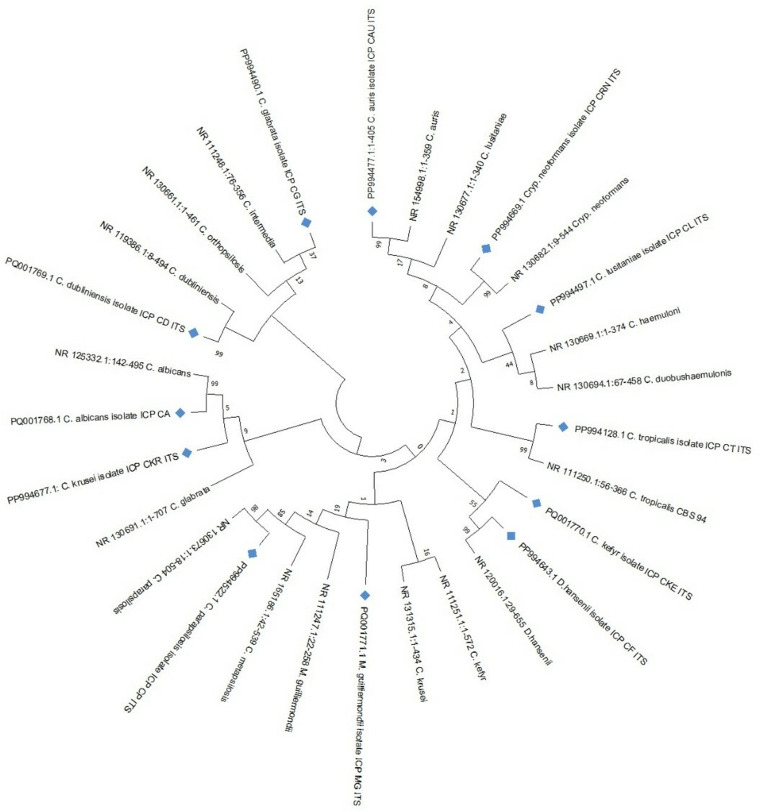
Phylogenetic tree of yeast species constructed using MEGA software.

The aligned sequences were downloaded in FASTA format and further processed in MEGA software alongside the query sequences. Phylogenetic trees were constructed using Maximum Likelihood Model, enabling the identification of yeast species based on sequence homology (Fig 14).

#### Validation of yeasts identification through MALDI-TOF.

The validation process, conducted using VITEK and MALDI-TOF analyses, successfully confirmed the accurate identification tool, generated detailed biochemical profiles that corresponded to specific yeast species. Similarly, MALDI-TOF (Matrix-Assisted Laser Desorption/Ionization Time-of-Flight) mass spectrometry produced unique protein spectra, enabling precise identification through characteristic protein signatures. The results from both methods demonstrated strong concordance, emphasizing the reliability of these complementary techniques for yeast identification. This validation underscores their importance in microbiological studies, ensuring consistent and reproducible results across diverse yeast samples.

### Statistical analysis

Descriptive crosstab analysis, binary logistic and multinomial logistic regression analyses were applied to evaluate the effects of various variables on the occurrence of different types of yeast infections. The values of key findings are summarized in Table 8.

**Table 8:** Summarized values of key (significant) findings of crosstab analysis, binary and multinomial regression analysis.

Crosstab analysis:								
Characteristics		Number	Culture test	Chi-square	P-value	95%Cl	Asymp. std. error	
			+ve	‐ve				
Age		608	256	352	108.711	0.237	0.241–0.258	0.040
Ward		608	451	157	33.725	0.000	0.000–0.000	0.037
Sample type		608	256	352	104.397	0.000	0.000–0.000	0.040
**Health disorder**								
Hypertension		309	131	178	20.018	0.045	0.028–0.035	0.041
Chest Infection		184	95	89	29.987	0.002	0.000–0.001	0.040
**Symptoms**								
Ear Itching		76	35	41	22.862	0.018	0.023–0.029	0.037
**Binary regression analysis (model summary):**								
Step	-2 Log likelihood				Cox & Snell R square		Nagelkarke R square	
1	150.185^a^				0.672		0.903	
**Binary regression** a**nalysis (output of variables in equation):**								
**Step 1**^a^: **independent variables**	**B**	**S.E.**			**Wald**	**df**	**Sig.**	**Exp(B)**
Ward	2.891	.551			27.488	1	.000	18.002
Sample Type	-6.448	.803			64.438	1	.000	.002
Constant	20.342	5.179			15.426	1	.000	682778563.595
**Multinomial regression** a**nalysis:**								
**Effects** **(independent variables)**		**Model fitting criteria**			**Likelihood ratio tests**			
		**-2 log likelihood of reduced model**			**Chi-Square**		**df**	**Sig.**
Ward		1378.932^c^			44.499		12	.000
Hypertension		1359.792^c^			25.359		12	.013
Meningitis		1334.433^b^			.000		0	
Sample type		1474.729^c^			140.296		36	.000

^a^Estimation terminated at iteration number 9 because parameter estimates changed by less than.001.

^b^This reduced model is equivalent to the final model because omitting the effect does not increase the degrees of freedom.

^c^Unexpected singularities in the Hessian matrix are encountered. This indicates that either some predictor variables should be excluded or some categories should be merged.

Crosstab analysis showed a significant relationship between types of yeast species vs. different dependent variable, i.e., age (70–79), ward, gender, health disorders (hypertension and chest infection) and symptoms (ear itching). Analysis through binary logistic regression demonstrated that the model was highly suitable (Nagelkarke, R^2^ value 0.903) where the yeast infection was 90.3% explained by independent variables (age, gender, wards, sample type, health disorder and symptoms).

The model fitting information demonstrated that the multinomial logistic regression model was highly appropriate and effectively explaining the association between yeast infection and the independent variables (χ 536.226, df 360, significance p<0.000). The Goodness of Fit test further validated the model’s suitability, with the Pearson Chi-square value being highly significant (0.000, i.e., >0.05). The R^2^ values for Cox & Snell (0.586) and Nagelkarke (0.613) indicated that the independent variables explained 58.6% and 61.3% of the variance in yeast species identification, respectively as summarized in Table 8 (the details are attached as S1 Data).

### Antifungal susceptibility testing (AST)

The antifungal susceptibility of 11 different *Candida* species was accessed using YM agar. *Candida auris* exhibited resistance to all three tested antifungal drugs. Among the drugs tested, Fluconazole showed the highest resistance rates, while Amphotericin-B was the most effective against the *Candida* species. *C. albicans* and *C. krusei* demonstrated high susceptibility to all three drugs. *Meyerozyma* (*Candida*) *guilliermondii* was particularly susceptible to Voriconazole, while Amphotericin-B was most effective against *C. lusitaniae. C*. *parapsilosis* exhibited susceptibility to all three tested antifungal drugs. A summary of drug resistance pattern is provided in Table 9, and corresponding figures (Figs 15–25) are attached as S2 Data.

**Table 9:** Antifungal susceptibility testing of yeast species against different drugs used in the study.

No.	Yeast *spp*.	Concentration of antifungal drug in µg/ml and zone of inhibition in millimeter (mm)^*^														
		Fluconazole					Amphotericin-B					Voriconazole				
		05	10	15	20	25	0.5	1.0	2.0	3.0	4.0	0.5	1.0	2.0	3.0	4.0
**1.**	*Candida albicans*	–	11.6	21.0	32.0	40.0	–	–	–	–	13.6	–	–	–	–	24.2
**2.**	*C*. *auris*	–	–	–	–	–	–	–	–	–	–	–	–	–	–	–
**3.**	*C*. *dubliniensis*	–	–	–	12.0	15.0	–	–	–	–	60.0	–	–	–	–	15
**4.**	*C*. *famata*	–	–	–	30.0	38.0	–	–	–	–	25.0	–	–	–	–	–
**5.**	*C*. *glabrata*	–	–	–	22.0	35.0	–	–	–	–	40.0	–	–	–	–	35.0
**6.**	*C*. *kefyr*	–	–	–	17.7	34.6	–	–	–	–	33.0	–	–	–	–	42.0
**7.**	*C. krusei*	6.0	13.7	14.0	21.0	41.0	–	–	60	15.0	38.0	–	–	–	15.0	45.0
**8.**	*C*. *lusitaniae*	–	–	11.2	18.6	21.4	–	11.5	13.1	14.7	16.2	–	–	–	15.3	25.6
**9.**	*C*. *parapsilosis*	–	–	–	–	46.7	–	–	–	12.5	52.4	–	–	–	–	33.5
**10.**	*C*. *tropicalis*	–	–	–	–	31.0	–	–	–	–	41.3	–	–	11.5	15.2	26.4
**11.**	*Meyerozyma* (*Candida*) *guilliermondii*	–	–	–	–	–	–	–	–	20.3	58.5	10.4	12.7	18.2	26.4	78.9

*– shows that no zone of inhibition was produced.

## Discussions

Most yeast cultures in this study formed glossy colonies with smooth edges, displaying colors ranging from white to cream and pink after 48 hours of incubation at 37°C on YM agar. Saliva samples yielded the highest number of positive cultures, followed by ear samples [56]. Colonies with white and cream hues were predominantly associated with the *Candida* genus, prompting further analysis using chromogenic media such as Brilliance *Candida* Agar (BCA) to differentiate between *Candida* species [57–58].

BCA effectively identified species such as *C. albicans, C. dubliniensis, C. krusei, C. tropicalis*, and *C. parapsilosis*. However, identifying *C. auris* proved challenging due to its variable colony colors, including beige, cream, and pink. While BCA successfully distinguished *C. tropicalis* and *C. dubliniensis*, the differentiation of *C. albicans and C. glabrata* was less definitive [16,59]. Additionally, some cultures were not identifiable using BCA and CCP, highlighting the limitations of these methods [15,59].

These limitations prompted a shift toward molecular methods for yeast identification. The ITS regions of fungal ribosomal DNA (rDNA), located between the 18S, 5.8S, and 28S coding regions, are commonly used for PCR-based fungal species differentiation. These regions are highly conserved yet evolve slowly, making them ideal targets for identification. Reference sequences for these regions are available in the NCBI database [18,20]. In this study, PCR amplification of the yeast ITS regions was performed using primer ITS1–4, ITS1–2, and ITS3–4 producing fragments between 148 and 872 bp which were visualized on an agarose gel [60]. Further species identification was achieved through species-specific PCR targeting genes such as PLB, Topoisomerase II, CDR, and 18S, along with PCR-RFLP analysis using the *Msp*I enzyme [61]. These techniques provide accessible alternatives for routine diagnostics laboratories that often lack resources for DNA sequencing.

In the RFLP assay using the *Msp*1 restriction enzyme on ITS PCR-amplified products, 12 species, including *Candida albicans, C. dubliniensis, C. krusei, C. glabrata, C. lusitaniae, C. tropicalis,* and *Cryptococcus neoformans*, produced two fragments, while *C. guilliermondii* yielded three fragments, corroborating prior findings [62]. However, *C. auris*, *C. famata, C. kefyr,* and *C. parapsilosis* did not generate digested products, further confirming earlier reports [63–64].

The Phospholipase-B (PLB) gene, essentials for maintaining membrane stability and nutrient uptake, has fewer variable regions than rRNA genes, making it a reliable marker for accurate species identification [21,37,65]. DNA topoisomerases, which regulate DNA topology, are critical targets for pharmacological intervention. Despite limited research on fungal topoisomerase II, this gene was selected due to its species-specific sequence. [20,34,66,67]. Primers targeting topoisomerase II gene variation were designed for *C. auris, C. lusitaniae, C. glabrata,* and *C. parapsilosis*, producing PCR bands of 209 bp, 154 bp, 220 bp, and 219 bp, respectively.

Similarly, *Candida* Drug Resistance (CDR) genes, primarily studied for their role in antifungal resistance, have been underutilized for species identification. In this study, species-specific primers targeting the CDR1 gene were employed to identify *C. auris*, *C. glabrata*, *C. dubliniensis*, *C. parapsilosis*, and *C. tropicalis*, yielding the expected PCR bands of 161 bp, 188 bp, 241 bp, 221 bp, and 163 bp, respectively.

DNA sequencing of the ITS region confirmed the accurate identification of all yeast species analyzed in the study. Genetic relatedness among these species was evaluated by constructing a phylogenetic tree using Maximum Likelihood (ML) analysis in MEGA11 software, based on DNA sequence data obtained through BLAST comparisons [68–72]. The analysis revealed substantial homogeneity among the isolates and identifying 11 species of *Candida* and one species of pink yeast, comprising *C. albicans*, *C. auris*, *C. dubliniensis*, *C. famata*, *C. glabrata*, *C. guilliermondii*, *C. kefyr*, *C. krusei*, *C. lusitaniae*, *C. parapsilosis*, *C. tropicalis*, and *Cryptococcus neoformans*.

The antifungal susceptibly of these yeast species was accessed using three antifungal agents: fluconazole, amphotericin-B, and voriconazole. *Candida auris* exhibited resistance to all three agents, with fluconazole showing the highest resistance, while amphotericin-B emerged as the most effective antifungal. *C. albicans* displayed the greatest susceptibility to all three drugs. Additionally, *Meyerozyma* (*Candida*) *guilliermondii* showed significant susceptibility to voriconazole, and amphotericin-B demonstrated the highest efficacy against *C. lusitaniae*. Meanwhile, *C. parapsilosis* was found to be susceptible to all three antifungal agents [73].

## Conclusion

This study is the first of its kind in Quetta, Balochistan, to document the presence of *Candida auris* among immunocompromised patients in special care and intensive care units. The detection of various yeast infections in immunocompromised patients can be enhanced through the application of diverse diagnostic techniques. Using of species-specific primers targeting various genes proved to be a rapid method for identifying the human pathogenic yeasts studied.

Statistical analyses, including crosstab, binary and multinomial logistic regression, revealed significant associations between yeast infection and various factors.

The study also highlighted antifungal resistance in certain *Candida* species, particularly against fluconazole, whereas amphotericin B was identified as the most effective antifungal agent.

Future research will focus on developing species-specific primers and probes for real-time PCR to target additional genes, thereby improving the identification of *Candida auris* and other *Candida* species. Researchers will also explore the role of the *Candida* drug resistance (*CDR1*) gene in contributing to drug resistance across various *Candida* species and antifungal drugs. Furthermore, antifungal susceptibility testing will be expanded to include echinocandin-class medications and other available therapies to identify more effective treatment options.

### Limitations of study

“The present study provides significant insights into the prevalence, identification, and antifungal resistance of *Candida auris* in immunocompromised patients in Quetta, Balochistan; however, certain limitations were identified. The broth microdilution assay could not be employed for antifungal susceptibility testing due to a lack of resources, including specialized equipment, and reagents etc. Additionally, echinocandin drugs were not included in antifungal susceptibility testing due to their unavailability in the region. Limited resources and inadequate funding posed significant challenges to conducting research in this underdeveloped area, hindering access to essential equipment, reagents, and infrastructure necessary for advanced scientific studies.”

## Supporting information

S1 Data Detailed results of statistical analysis. (DOCX)

S2 Data Images of antifungal susceptibility testing. (DOCX)

S1 Fig Raw images. (PDF)
