# Supplementary material for: Investigation of multi-drug resistant Candida auris using species-specific molecular markers in immunocompromised patients from a tertiary care hospital in Quetta, Pakistan
Source: PLoS One. 2025 Apr 24;20(4):e0319485. doi: 10.1371/journal.pone.0319485 (PMC12021172; doi:10.1371/journal.pone.0319485)
Supplement: S1 Data — (DOCX) [file pone.0319485.s001.docx]

**Supporting Information (SI_1): Detailed Results of Statistical Analysis**

**Appendix 1:** Descriptive Analysis of Frequency Distribution by Crosstab Analysis

| **Characteristics** | **Number** | **Culture Test** | | **Chi-square** | **P-value** | **95%Cl** | **Asymp.**  **Std. Error** |  |
| --- | --- | --- | --- | --- | --- | --- | --- | --- |
|  |  | **+ve** | **-ve** |  |  |  |  |  |
| **Age in Years** | | | | | | | |  |
| 01-09  10-19  20-29  30-39  40-49  50-59  60-69  70-79  80-89  90-99 | 5 | 1 | 4 | 2.889 | 0.992 | 0.900-0.915 | 0.045 |  |
|  | 24 | 8 | 16 | 9.500 | 0.576 | 0.451-0.470 | 0.041 |  |
|  | 37 | 16 | 21 | 6.231 | 0.858 | 0.795-0.810 | 0.037 |  |
|  | 12 | 8 | 4 | 17.422 | 0.096 | 0.124-0.137 | 0.036 |  |
|  | 60 | 25 | 35 | 14.794 | 0.192 | 0.205-0.221 | 0.045 |  |
|  | 123 | 56 | 67 | 17.869 | 0.085 | 0.071-0.082 | 0.042 |  |
|  | 148 | 61 | 87 | 12.905 | 0.300 | 0.292-0.310 | 0.040 |  |
|  | 139 | 59 | 80 | 20.297 | 0.041 | 0.035-0.043 | 0.040 |  |
|  | 52 | 20 | 32 | 16.154 | 0.136 | 0.161-0.176 | 0.037 |  |
|  | 8 | 2 | 6 | 6.089 | 0.867 | 0.606-0.625 | 0.036 |  |
| Total | 608 | 256 | 352 | 108.711 | 0.237 | 0.241-0.258 | 0.040 |  |
| **Gender** | | | | | | | |  |
| Male  Female | 265 | 101 | 164 | 7.381 | 0.767 | 0.786-0.802 | 0.040 |  |
|  | 343 | 155 | 188 | 7.381 | 0.767 | 0.788-0.804 | 0.040 |  |
| Total | 608 | 256 | 352 | 7.381 | 0.767 | 0.788-0.804 | 0.040 |  |
| **Ward** | | | | | | | |  |
| ICU  SCU | 451 | 217 | 234 | 33.725 | 0.000 | 0.000-0.000 | 0.037 |  |
|  | 157 | 39 | 118 | 33.725 | 0.000 | 0.000-0.001 | 0.037 |  |
| Total | 608 | 451 | 157 | 33.725 | 0.000 | 0.000-0.000 | 0.037 |  |
| **Sample Type** | | | | | | | |  |
| Axilla  Ear  Groin  Saliva | 150 | 42 | 108 | 21.879 | 0.025 | 0.018-0.024 | 0.041 |  |
|  | 153 | 72 | 81 | 7.275 | 0.776 | 0.787-0.802 | 0.040 |  |
|  | 150 | 35 | 115 | 37.436 | 0.000 | 0.000-0.000 | 0.034 |  |
|  | 155 | 107 | 48 | 72.841 | 0.000 | 0.000-0.000 | 0.041 |  |
| Total | 608 | 256 | 352 | 104.397 | 0.000 | 0.000-0.000 | 0.040 |  |
| **Health Disorder** | | | | | | | |  |
| Diabetes  Hypertension  Covid-19  Chest Infection  Ear Infection  Heart Diseases  Pneumonia  Asthma  Tuberculosis Treated | 301 | 124 | 177 | 10.078 | 0.523 | 0.519-0.538 | 0.041 |  |
|  | 309 | 131 | 178 | 20.018 | 0.045 | 0.028-0.035 | 0.041 |  |
|  | 159 | 49 | 110 | 16.354 | 0.128 | 0.110-0.122 | 0.037 |  |
|  | 184 | 95 | 89 | 29.987 | 0.002 | 0.000-0.001 | 0.040 |  |
|  | 131 | 58 | 73 | 11.584 | 0.396 | 0.378-0.397 | 0.041 |  |
|  | 53 | 19 | 34 | 5.118 | 0.925 | 0.902-0.913 | 0.042 |  |
|  | 32 | 12 | 20 | 7.103 | 0.791 | 0.695-0.713 | 0.044 |  |
|  | 50 | 19 | 31 | 5.717 | 0.86 | 0.854-0.867 | 0.041 |  |
|  | 43 | 16 | 27 | 8.999 | 0.623 | 0.519-0.538 | 0.042 |  |
| **Symptoms** | | | | | | | |  |
| Fever  Chills  Ear Itching  Ear Pain  Blood in Sputum  Weakness  Shortness of Breath | 572 | 242 | 330 | 19.342 | 0.055 | 0.103-0.115 | 0.042 |  |
|  | 329 | 136 | 193 | 13.801 | 0.244 | 0.218-0.235 | 0.040 |  |
|  | 76 | 35 | 41 | 22.862 | 0.018 | 0.023-0.029 | 0.037 |  |
|  | 123 | 53 | 70 | 6.777 | 0.817 | 0.810-0.825 | 0.039 |  |
|  | 98 | 31 | 67 | 11.655 | 0.390 | 0.371-0.390 | 0.039 |  |
|  | 468 | 189 | 279 | 12.696 | 0.314 | 0.293-0.311 | 0.041 |  |
|  | 318 | 124 | 194 | 8.536 | 0.665 | 0.671-0.689 | 0.041 |  |

**Appendix 2:** Model Summary of Binary Regression Analysis

| Step | -2 Log likelihood | Cox & Snell R Square | Nagelkarke R Square |
| --- | --- | --- | --- |
| 1 | 150.185^a^ | 0.672 | 0.903 |
| a. Estimation terminated at iteration number 9 because parameter estimates changed by less than .001. | | | |

**Appendix 3:** Output of Variables in Equation of Binary Logistic Regression

| 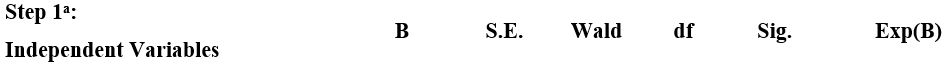 | | | | | | |
| --- | --- | --- | --- | --- | --- | --- |
| Ward | 2.891 | .551 | 27.488 | 1 | .000 | 18.002 |
| Gender | -.102 | .459 | .049 | 1 | .824 | .903 |
| Age Groups | -.003 | .125 | .001 | 1 | .982 | .997 |
| Fever | 1.694 | .935 | 3.283 | 1 | .070 | 5.442 |
| Chills | -.072 | .483 | .022 | 1 | .882 | .931 |
| Ear Infection | -.189 | .539 | .122 | 1 | .726 | .828 |
| Ear Itching | .025 | .715 | .001 | 1 | .972 | 1.025 |
| Ear Pain | .334 | .578 | .334 | 1 | .563 | 1.397 |
| Chest Infection | .158 | .558 | .080 | 1 | .778 | 1.171 |
| Blood in Sputum | -.472 | .616 | .587 | 1 | .444 | .624 |
| Weakness | -.505 | .650 | .602 | 1 | .438 | .604 |
| Diabetes | -.859 | .607 | 2.004 | 1 | .157 | .424 |
| hypertension | .315 | .580 | .296 | 1 | .586 | 1.371 |
| Covid-19 | -.688 | .561 | 1.503 | 1 | .220 | .503 |
| Shortness of Breath | -.870 | .539 | 2.605 | 1 | .107 | .419 |
| Heart Diseases | -.501 | .817 | .376 | 1 | .540 | .606 |
| Pneumonia | -.618 | .949 | .424 | 1 | .515 | .539 |
| Asthma | .012 | .899 | .000 | 1 | .989 | 1.012 |
| Tuberculosis Treated | -.402 | .832 | .233 | 1 | .629 | .669 |
| Sample Type | -6.448 | .803 | 64.438 | 1 | .000 | .002 |
| Constant | 20.342 | 5.179 | 15.426 | 1 | .000 | 682778563.595 |

**Appendix 4:** Likelihood Ratio Tests of Multinomial Logistic Regression

| 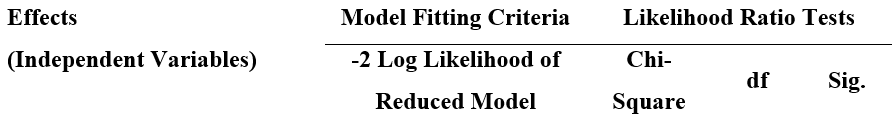 | | | | |
| --- | --- | --- | --- | --- |
| Intercept | 1334.433^a^ | .000 | 0 | . |
| Ward | 1378.932^b^ | 44.499 | 12 | .000 |
| Gender | 1340.572^b^ | 6.139 | 12 | .909 |
| Fever | 1353.502^b^ | 19.070 | 12 | .087 |
| Chills | 1345.561^b^ | 11.128 | 12 | .518 |
| Ear Infection | 1338.662^b^ | 4.229 | 12 | .979 |
| Ear Itching | 1354.069^b^ | 19.636 | 12 | .074 |
| Ear Pain | 1338.127^b^ | 3.694 | 12 | .988 |
| Chest Infection | 1348.865^b^ | 14.432 | 12 | .274 |
| Blood in Sputum | 1345.352^b^ | 10.920 | 12 | .536 |
| Weakness | 1337.903^b^ | 3.471 | 12 | .991 |
| Diabetes | 1349.457^b^ | 15.024 | 12 | .240 |
| hypertension | 1359.792^b^ | 25.359 | 12 | .013 |
| Covid-19 | 1347.716^b^ | 13.283 | 12 | .349 |
| Shortness of Breath | 1346.047^b^ | 11.614 | 12 | .477 |
| Heart Diseases | 1339.319^b^ | 4.886 | 12 | .962 |
| Pneumonia | 1340.429^b^ | 5.996 | 12 | .916 |
| Asthma | 1339.672^b^ | 5.239 | 12 | .950 |
| Tuberculosis Treated | 1348.800^b^ | 14.367 | 12 | .278 |
| Meningitis | 1334.433^a^ | .000 | 0 | . |
| Sample Type | 1474.729^b^ | 140.296 | 36 | .000 |
| Age Groups | 1451.387^b^ | 116.955 | 108 | .262 |

**a.)** This reduced model is equivalent to the final model because omitting the effect does not increase the degrees of freedom. **b.)** Unexpected singularities in the Hessian matrix are encountered. This indicates that either some predictor variables should be excluded or some categories should be merged.
