# Supplementary material for: Investigation of multi-drug resistant Candida auris using species-specific molecular markers in immunocompromised patients from a tertiary care hospital in Quetta, Pakistan
Source: PLoS One. 2025 Apr 24;20(4):e0319485. doi: 10.1371/journal.pone.0319485 (PMC12021172; doi:10.1371/journal.pone.0319485)
Supplement: S2 Data — (DOCX) [file pone.0319485.s002.docx]

**Supporting Information (SI 2):** Images of Antifungal Susceptibility Testing


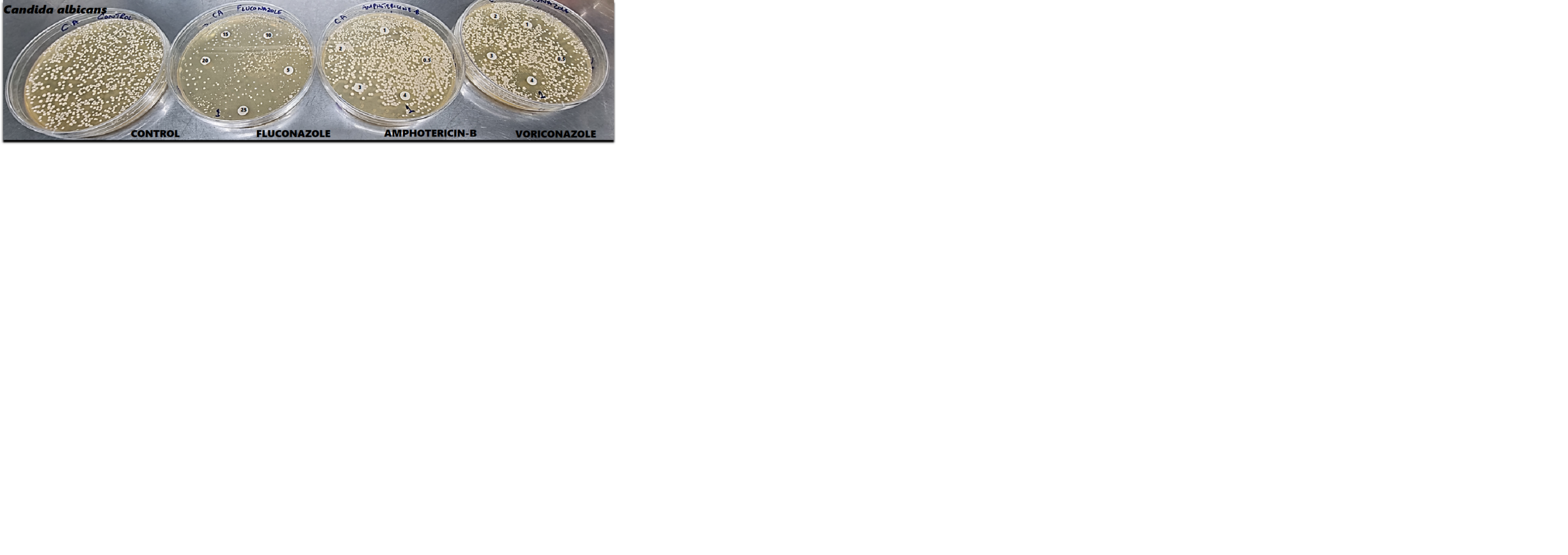


**Fig 15** AST of *Candida albicans* with Fluconazole, Amphotericin-B, and Voriconazole Drugs.


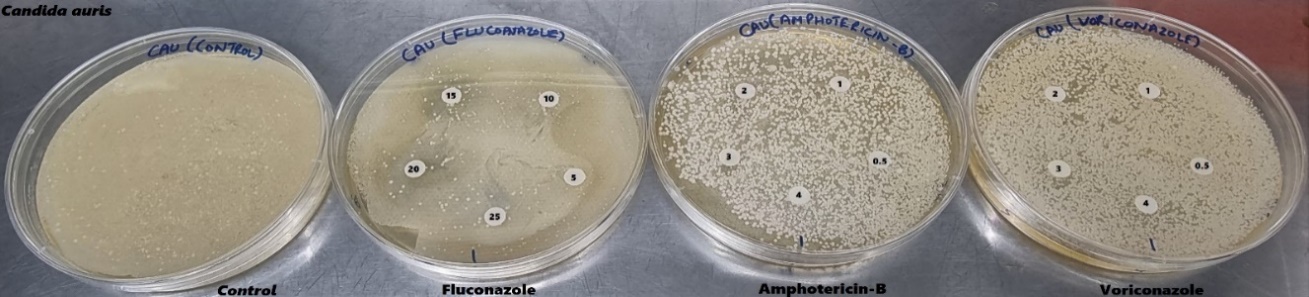


**Fig 16** AST of *C. auris* with Fluconazole, Amphotericin-B and Voriconazole Drugs


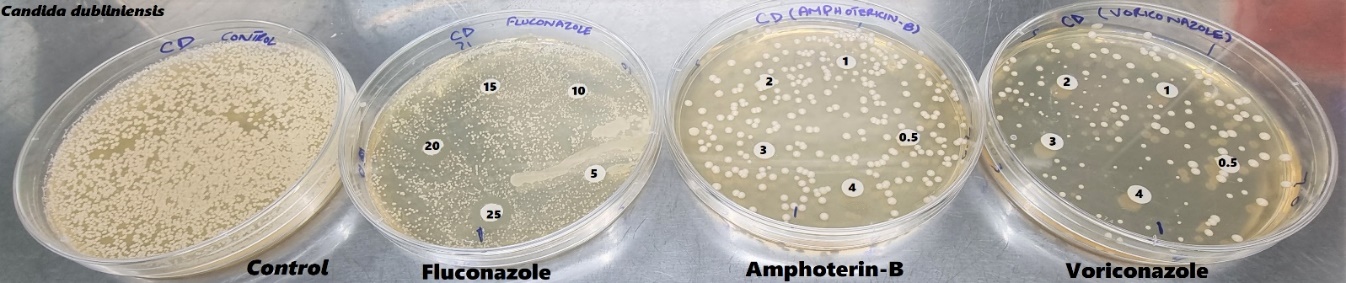


**Fig 17** AST of *C. dubliniensis* with Fluconazole, Amphotericin-B, and Voriconazole Drugs.


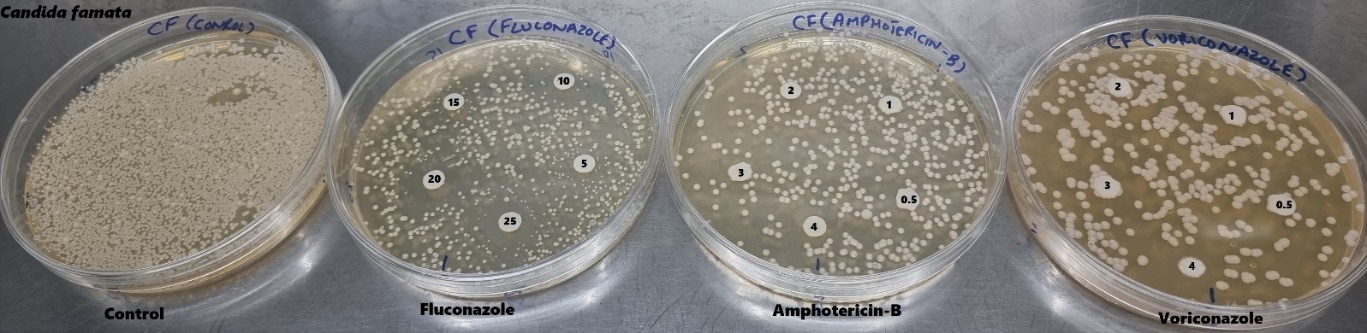


**Fig 18** AST of *C. famata* with Fluconazole, Amphotericin-B and Voriconazole Drugs


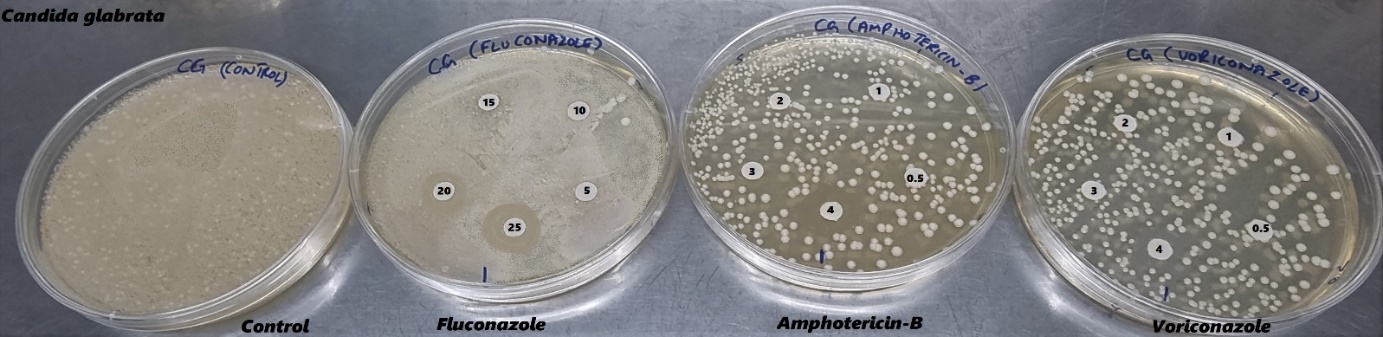


**Fig 19** AST of *C. glabrata* with Fluconazole, Amphotericin-B and Voriconazole Drugs


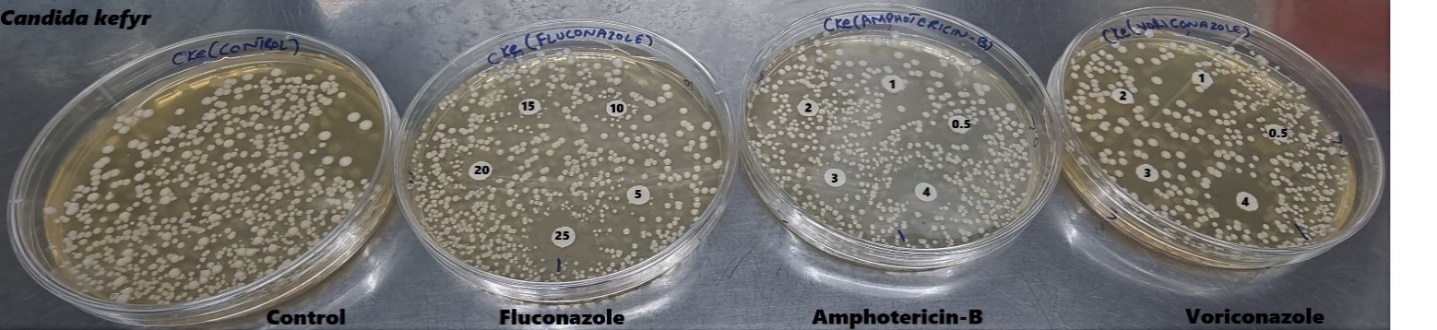


**Fig 20** AST of *C. kefyr* with Fluconazole, Amphotericin-B and Voriconazole Drugs


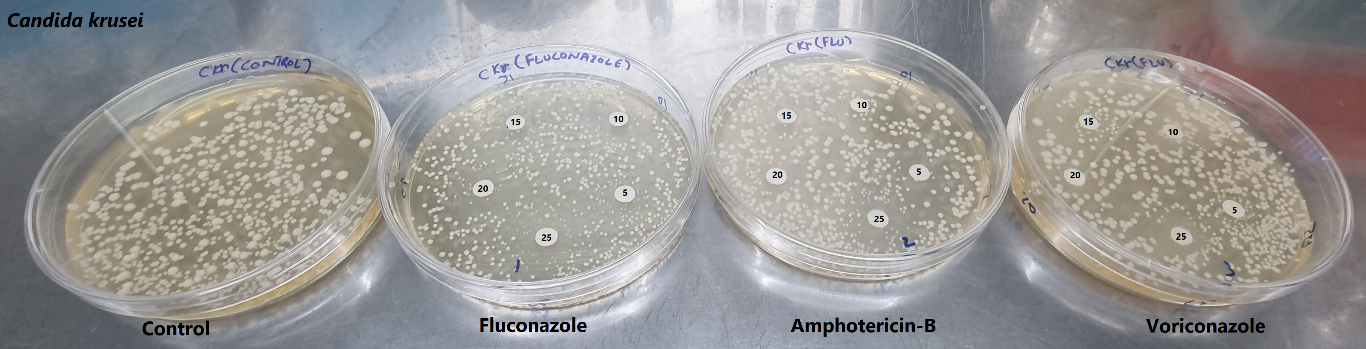


**Fig 21** AST of *C. krusei* with Fluconazole, Amphotericin-B and Voriconazole Drugs


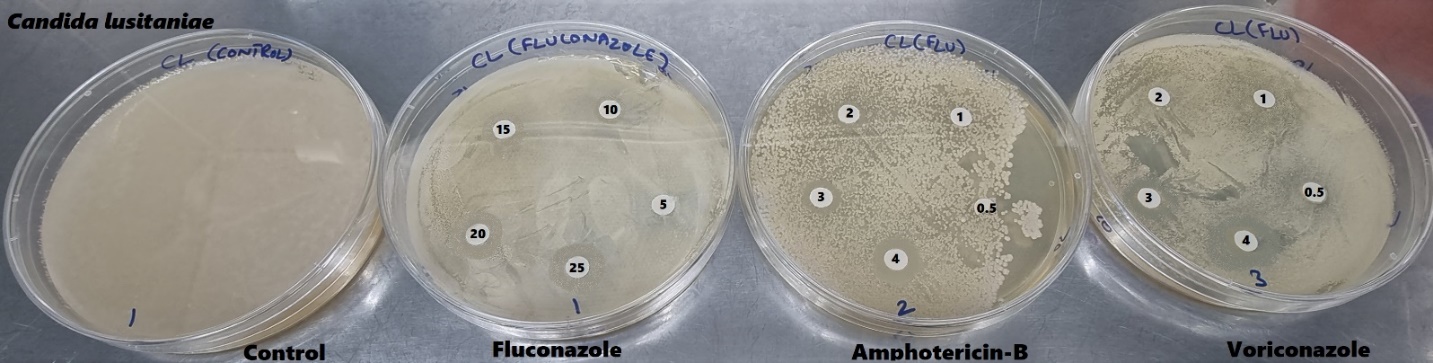


**Fig 22** AST of *C. lusitaniae* with Fluconazole, Amphotericin-B and Voriconazole Drugs


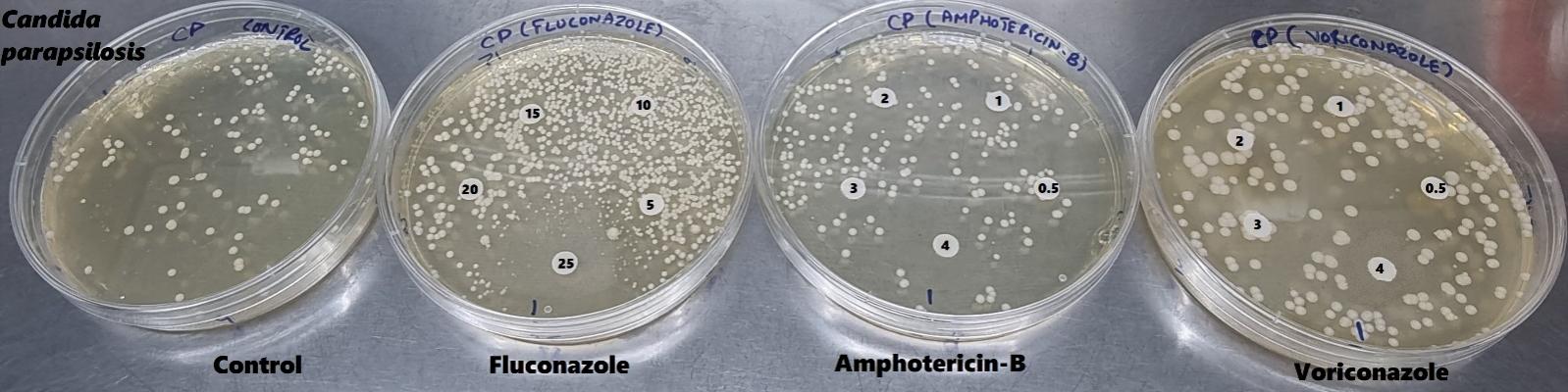


**Fig 23** AST of *C. parapsilosis* with Fluconazole, Amphotericin-B, and Voriconazole Drugs.


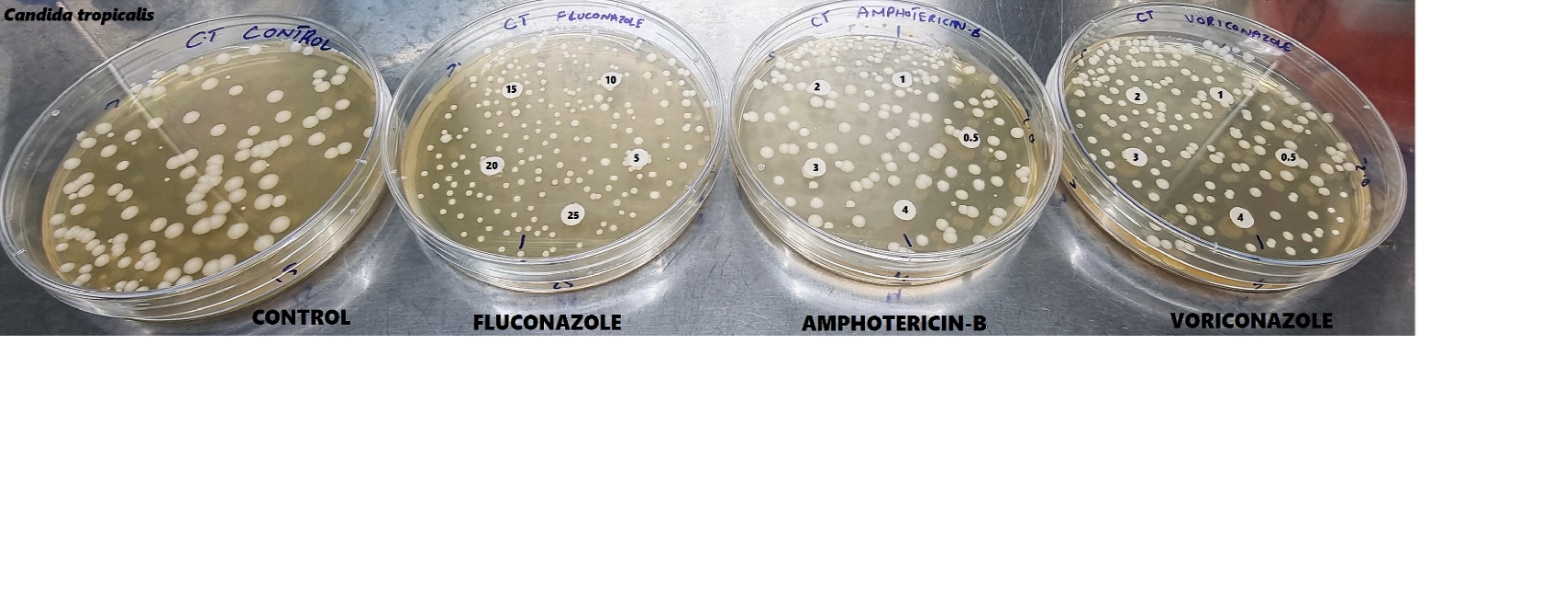


**Fig 24** AST of *C. tropicalis* with Fluconazole, Amphotericin-B, and Voriconazole Drugs.


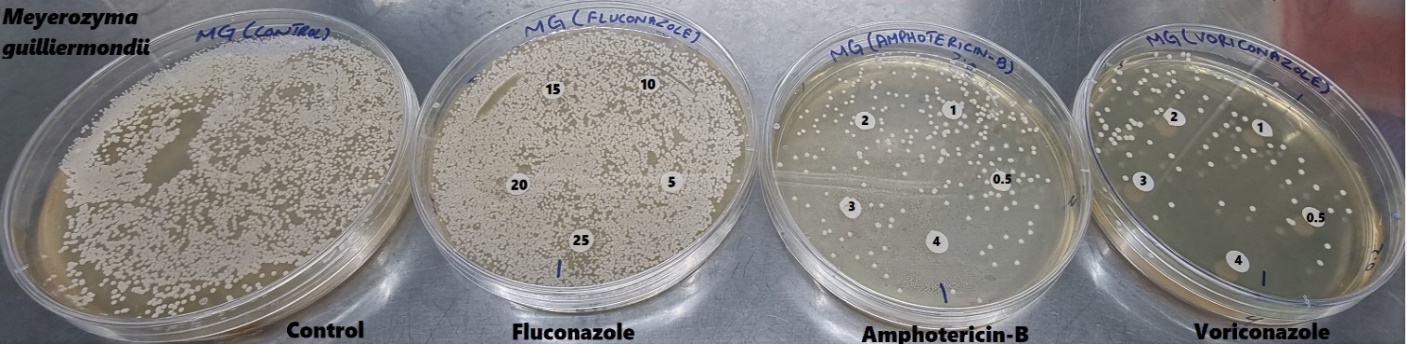


**Fig 25** AST of *Meyerozyma guilliermondii with* Fluconazole, Amphotericin-B and Voriconazole Drugs
