# Supplementary material for: Investigation of multi-drug resistant Candida auris using species-specific molecular markers in immunocompromised patients from a tertiary care hospital in Quetta, Pakistan
Source: PLoS One. 2025 Apr 24;20(4):e0319485. doi: 10.1371/journal.pone.0319485 (PMC12021172; doi:10.1371/journal.pone.0319485)
Supplement: S1 Fig — (PDF) [file pone.0319485.s003.pdf]

File Name: S1_raw images

(Uncropped and Unedited Raw Images of All PCR Amplified Products)

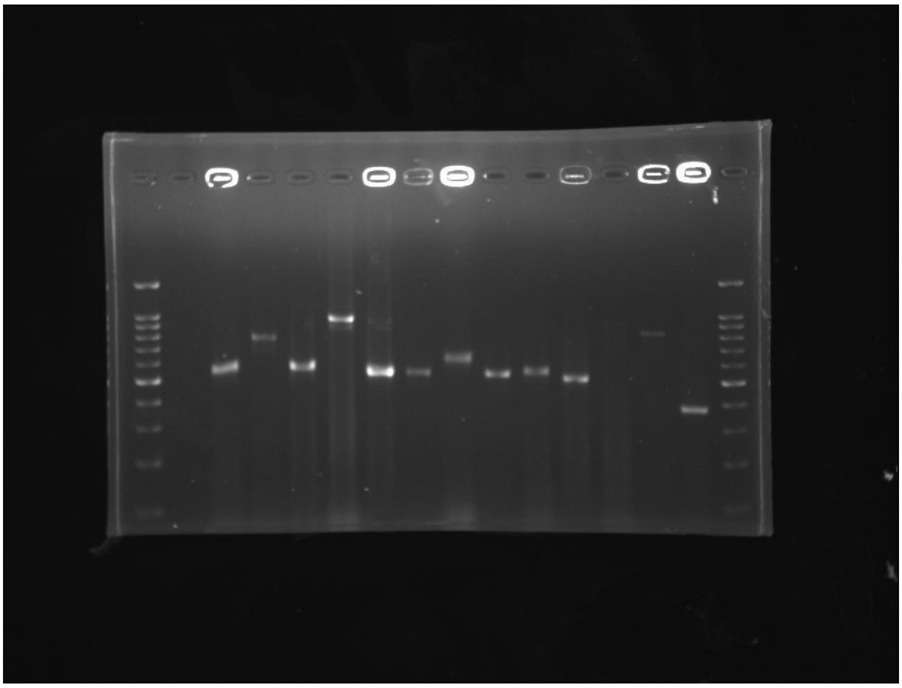

Figure 3: PCR amplified products using ITS1&4 primers of ITS regions of yeast species: 2= *C. albicans* (535bp), 3= *C. kefyr* (771bp), 4= *C. dubliniensis* (540bp), 5= *C. glabrata* (880bp), 6= *C. krusei* (510bp), 7=*C. parapsilosis* (520bp), 8= *C. famata* (633bp), 9= *C. tropicalis* (524bp), 10= *C. dubliniensis* (540 bp), 11= *C. krusei* (510bp), 12 shows no result, 13= *C. glabrata* (871bp), and 14= *C. lusitaniae* (377bp) as compared to negative control (1) and 100bp DNA ladder (D1 and D2).

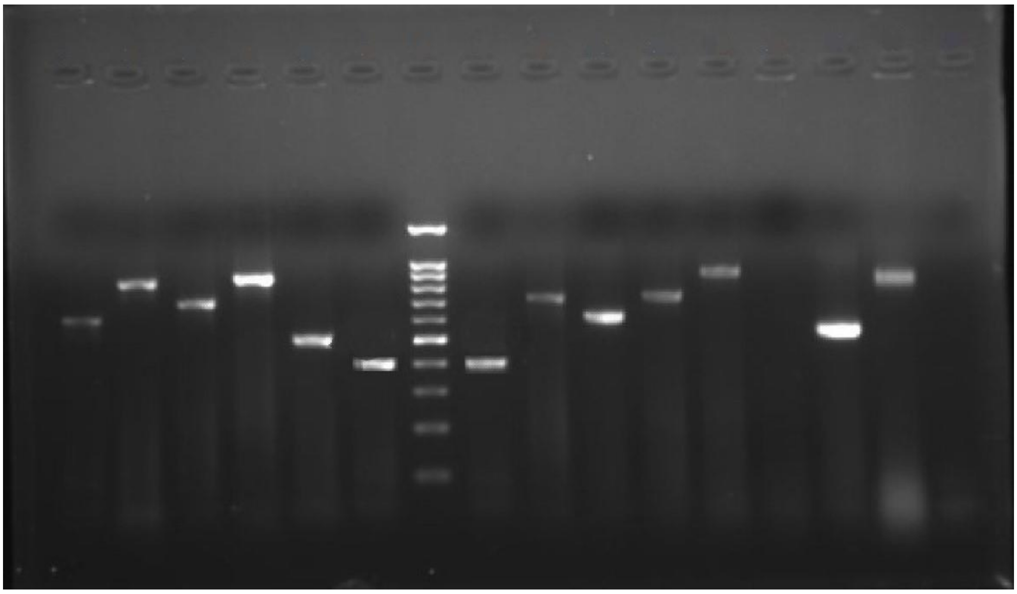

Figure 4: PCR amplified products of ITS1&4 primers of yeast species., 1= *C. glabrata* (871bp), 2= *C. kefyr* (771bp), 3= *C. glabrata* (871bp), 4= *C. albicans* (535bp), 5&7= *C. auris* (400bp), 8= *Meyerozyma guilliermondii* (607bp), and 6= 100bp DNA ladder

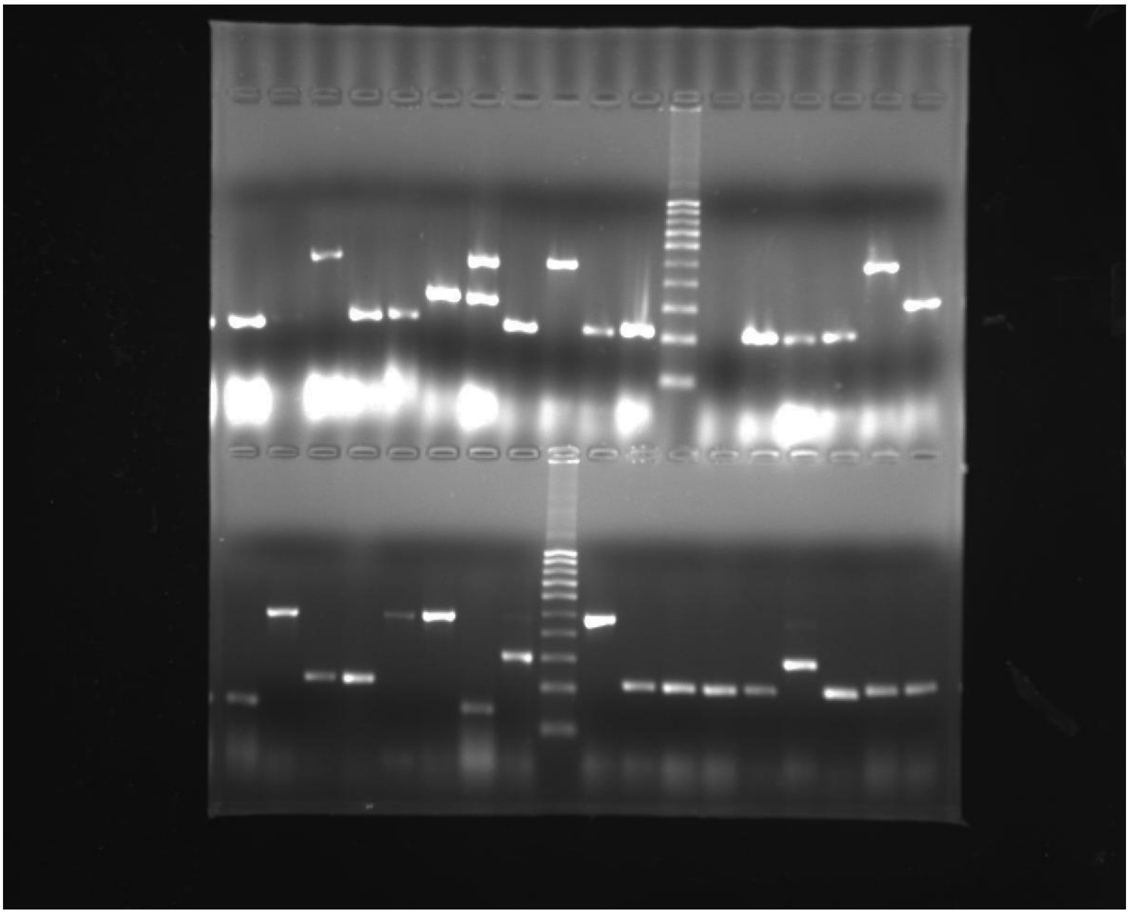

Figure 5: PCR amplified products using ITS1&2 primers of ITS regions of yeast spp., CAU= *Candida auris* (154bp), CG= *C. glabrata* (482bp), MG= *Meyerozyma guilliermondii* (248bp), CP= *C. parapsilosis* (229bp), 7= *C. lusitaniae* (148bp), 8= *C. kefyr* (309bp), 9= *C. albicans* (218bp),10= *C. dubliniensis* (218bp, 12= *Cryptococcus neoformans* (201bp) 13= *Candida famata* (278bp) and 14= *Candida tropicalis* (218bp)

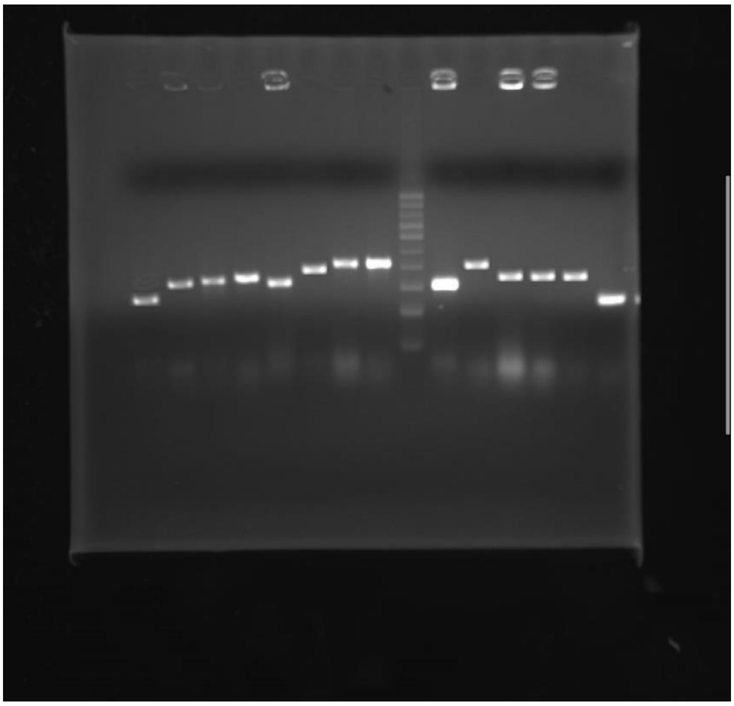

Figure 6: PCR amplified products using ITS3&4 primers of ITS regions of yeast species. CAU= *Candida auris* (267bp), CA= *C. albicans* (328bp), and CT=*C. tropicalis* (329bp), CD= *C. dubliniensis* (343bp), CP= *C. parapsilosis* (311bp), CF= *C. famata* (381bp), CKE= *C. kefyr* (432bp) CG= *C. glabrata* (419bp), CF= *C. famata* (381bp, CN= *Cryptococcus neoformans* (373bp) CL= *C. lusitaniae* (255bp) as compared to 100bp DNA ladder (9).

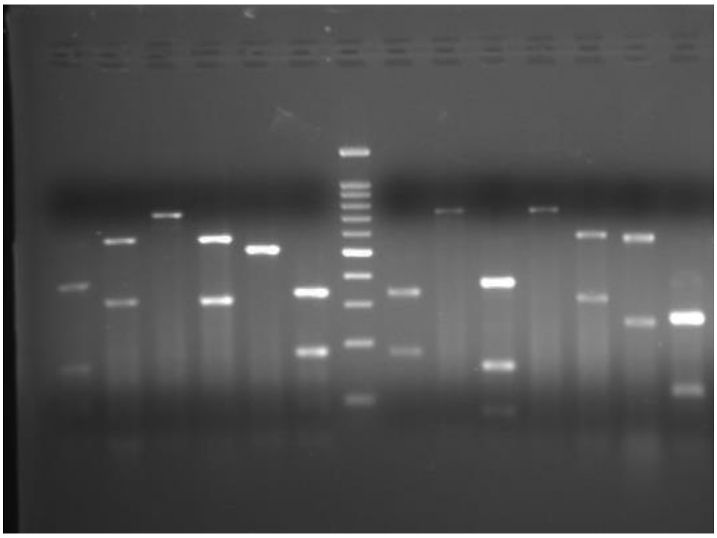

Figure 7: Gel image of PCR-RFLP Assay of ITS1&4 primers amplified products using Msp*1* as a Restriction enzyme. 1= *Candida tropicalis* (340,186bp), 2, 12, 13= *C. glabrata* (320, 561bp), 3, 9, 11= *C. kefyr* (771bp), 4= *C. parapsilosis* (530bp), 5= *C. dubliniensis* (340,200bp), 6= *C. dubliniensis* (340, 200bp), 7= *Meyerozyma guilliermondii* (82, 155, 370bp) 8= *C. lusitaniae* (250,120bp), and as compared to 100bp DNA ladder (7).

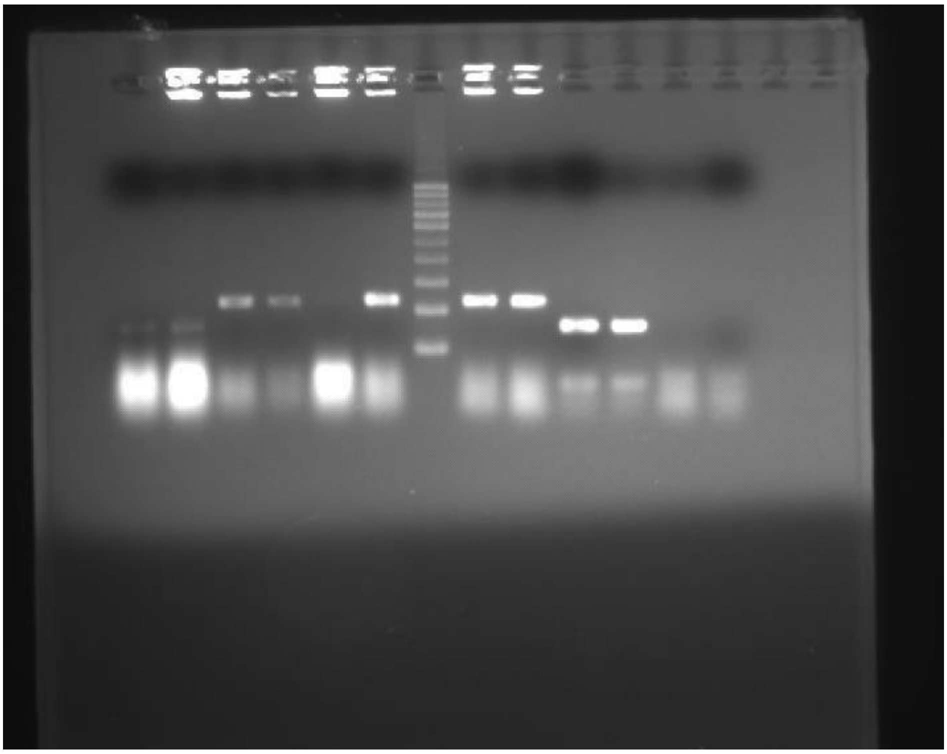

Figure 10: Using PLB gene species-specific primers PCR amplified product of CA=*C. albicans* (164bp), CT=*C. tropicalis* (230bp), *C. dubliniensis* (225bp), *C. parapsilosis* (234bp), *Meyerozyma guilliermondii* (161bp) and 100bp DNA ladder (D)

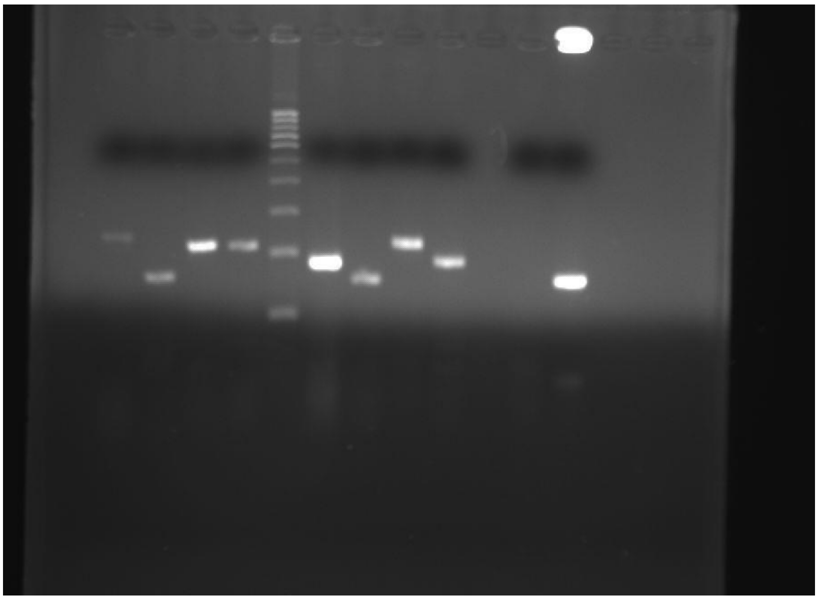

Figure 11: PCR amplified products of the PLB gene through specie-specific: CT = *Candida tropicalis* (230bp), CKE = *C. kefyr* (168bp), CP = *C. parapsilosis* (234bp), CD = *C. dubliniensis* (225bp), CF= *C. famata* (193bp), MG= *Meyerozyma guilliermondii* (161bp), CN= *Cryptococcus neoformans* (200bp) CL= *C. lusitaniae* (177bp) and D= 100bp ladder (5).

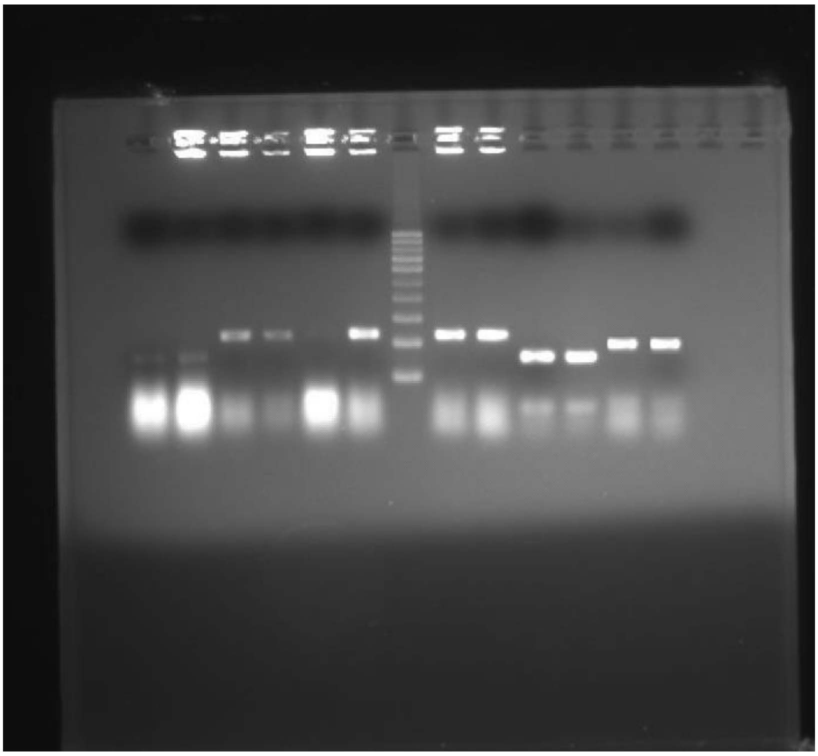

Figure 13: PCR amplified products of the *Candida auris* PLB= 230bp, CDR=164bp, ITS2= 167bp and TOP= 209bp using **PLB**, **CDR**, **ITS2** and **Topoisomerase II (TOP)** gene through specie specific primers.

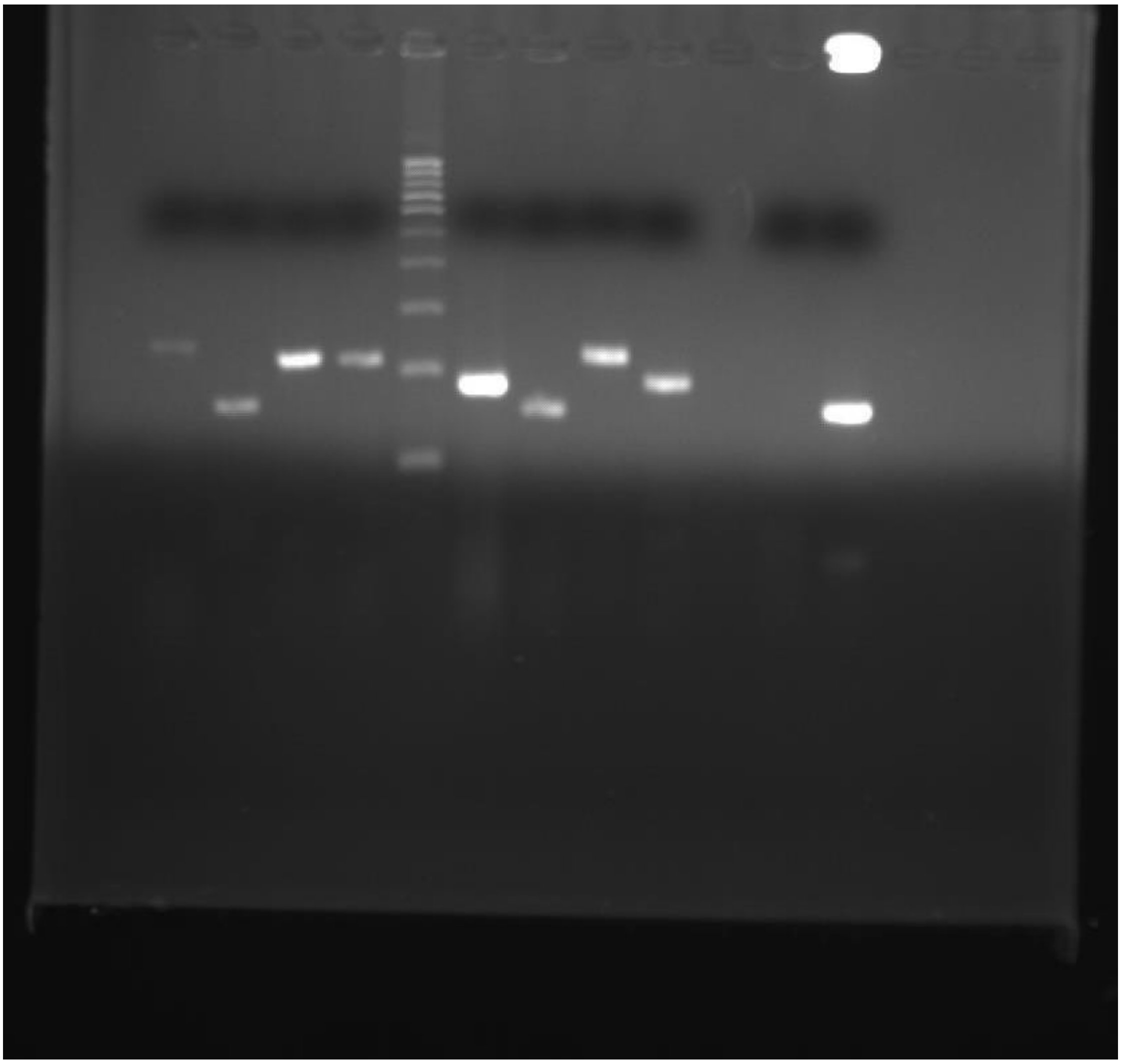

Figure 13: PCR amplified products through specie-specific primers of **Topoisomerase II gene:** CL(TOP) = *C. lusitaniae* (219bp), CP(TOP) = *C. parapsilosis* (220bp), CG(TOP) = *C. glabrata* (160bp); **CDR gene**: CD(CDR)= *C. dubliniensis* (241bp), CT(CDR)= *C. tropicalis* (163bp), CP(CDR)= *C. parapsilosis* (221bp), CG(CDR)= *C. glabrata* (188bp); and D=100bp ladder.
